# Oral Frailty and Multidimensional Health Among Community-Dwelling Older Adults in China: A Cross-Sectional Study

**DOI:** 10.3390/nu18142250

**Published:** 2026-07-09

**Authors:** Wenpeng Li, Shijun Tang, Lunrongyi Tian, Jianrui Zhai, Junchen He, Jiahui Li, Ziwen Zhao, Zhilu Zhou, Meihong Xu

**Affiliations:** 1 The School of Public Health, Peking University, Beijing 100191, China; 2210306221@stu.pku.edu.cn (W.L.); 2210306223@stu.pku.edu.cn (S.T.); 2210306224@stu.pku.edu.cn (L.T.); 2210306227@stu.pku.edu.cn (J.Z.); 2210306238@stu.pku.edu.cn (J.H.); 2210306212@stu.pku.edu.cn (J.L.); 2210306228@stu.pku.edu.cn (Z.Z.); 2210306229@stu.pku.edu.cn (Z.Z.); 2 Department of Nutrition and Food Hygiene, School of Public Health, Beijing Key Laboratory of Toxicological Research and Risk Assessment for Food Safety, Peking University, Beijing 100191, China; 3 Institute of Medical Technology, Peking University Health Science Center, Beijing 100019, China

**Keywords:** oral frailty, multidimensional health, nutrition, dietary diversity, healthy aging, community-dwelling older adults, WS/T 802–2022

## Abstract

**Background/Objectives**: Oral frailty is increasingly recognized as an age-related decline in oral function and a potential marker of broader health vulnerability. However, its association with multidimensional health within a standardized healthy aging framework remains insufficiently characterized. This study aimed to examine the association between oral frailty and multidimensional health among community-dwelling older adults in China using the WS/T 802–2022 healthy aging framework. **Methods**: This community-based cross-sectional study included 454 adults aged 60 years or older from Chenzhou, Hunan Province, China. Oral frailty was primarily assessed using the Oral Frailty Index-8 (OF-8), with the Screening Tool for Oral Frailty-6 (SOFT-6) used as an alternative definition. Overall, physical, mental, and social health were evaluated according to WS/T 802–2022. Associations were examined using ordinal regression models, supplemented by sensitivity and dose–response analyses. **Results**: Oral frailty was common, with prevalence estimates of 59.1% by OF-8 and 52.3% by SOFT-6. Compared with participants without oral frailty, those with OF-8-defined oral frailty were older and had lower body mass index, lower skeletal muscle mass index, lower grip strength, greater chronic disease burden, fewer remaining natural teeth, and lower overall and physical health scores. In the main adjusted model, OF-8-defined oral frailty was associated with lower odds of being in a better overall health category (odds ratio [OR] = 0.554, 95% confidence interval [CI]: 0.374–0.822). For physical health, threshold-specific estimates from partial proportional odds models were directionally consistent but did not reach statistical significance. The association with social health was nominal, whereas the association with mental health was not statistically significant. Across sensitivity analyses, estimates for overall health were directionally consistent, whereas domain-specific findings varied across alternative oral-frailty definitions and analytical approaches. In sensitivity analyses excluding participants with extreme total energy intake, the association with overall health remained statistically significant, whereas the associations with physical and social health were attenuated and no longer reached conventional statistical significance. Restricted cubic spline analyses showed approximately monotonic inverse associations between OF-8 score and overall, physical, and mental health scores, without evidence of significant nonlinearity. Oral frailty was highly prevalent and was associated with poorer multidimensional health, particularly overall health, among community-dwelling older adults. **Conclusions**: These findings suggest that oral frailty, assessed with OF-8, may be a simple, nutrition-relevant indicator that could help flag community-dwelling older adults for fuller multidimensional geriatric assessment. Because the design was cross-sectional and associations beyond overall health were inconsistent, the screening or predictive performance of oral frailty was not formally evaluated and requires confirmation in longitudinal, multicenter studies.

## 1. Introduction

Population aging is reshaping health systems worldwide. As life expectancy increases, the central challenge for public health is no longer simply to prolong survival, but to maintain functional ability, independence, and quality of life in later life [1]. The World Health Organization emphasized healthy aging in its 2015 World Report on Aging and Health as the process of developing and maintaining functional ability, which is shaped by intrinsic capacity, environments, and social participation [2]. China has one of the world’s largest and fastest-growing older populations [3]. According to the Seventh National Population Census, more than 264 million Chinese people were aged 60 years or older, accounting for 18.70% of the total population, and this proportion is projected to exceed 30% by approximately 2035 [4]. Accordingly, geriatric health management in China, as in many other rapidly aging societies, is shifting from a disease-centered model toward multidimensional assessment and early identification of functional vulnerability [5].

Oral health is increasingly recognized as an integral component of healthy aging, yet oral functional decline remains under-recognized in many community-based health systems [6]. Age-related changes in chewing, swallowing, salivary function, oral motor ability, and tooth retention may affect food choice, nutritional adequacy, speech, self-confidence, interpersonal communication, and social participation [7]. Oral frailty is an emerging geriatric concept that was initially proposed in Japan in 2014 [8]. It refers to an age-related, cumulative, and potentially reversible decline in oral function, representing a transitional state between healthy oral function and irreversible oral dysfunction [6,8]. Several screening tools have subsequently been developed or applied in community and clinical contexts, including the Oral Frailty Index-8 (OF-8), shorter oral frailty checklists, and the six-item screening tool for oral frailty (SOFT-6) [9,10,11]. Because oral frailty is closely related to nutrition, communication, social engagement, and quality of life, it should not be viewed merely as a localized oral problem [12,13,14]. Rather, it may serve as a simple and scalable marker of broader geriatric vulnerability and may share biological and functional pathways with sarcopenia, malnutrition, depressive symptoms, cognitive impairment, and other geriatric syndromes [15,16]. From a nutritional perspective, oral frailty may be particularly relevant because oral function is a prerequisite for adequate dietary intake in later life. Declines in mastication, swallowing, salivary secretion, tongue pressure, oral motor function, and tooth retention may restrict food choices and reduce the intake of hard-to-chew but nutrient-dense foods, including meat, vegetables, fruits, and nuts. These changes may contribute to lower dietary variety, inadequate protein and energy intake, micronutrient insufficiency, and increased risk of malnutrition. Recent epidemiological evidence has shown that poor oral function and oral frailty are associated with poorer dietary intake, lower dietary variety, and deteriorating nutritional status among community-dwelling older adults [12,13,14]. Therefore, oral frailty may represent not only localized oral functional decline, but also a nutrition-relevant geriatric condition that reflects vulnerability in diet quality and nutritional resilience.

Evidence has increasingly linked oral frailty or oral hypofunction to adverse health outcomes [17]. Prospective cohort studies from Japan have shown that oral frailty is associated with incident physical frailty, functional disability, and mortality among community-dwelling older adults [17,18,19]. Other studies have reported associations between oral functional decline and depressive symptoms, cognitive impairment, and social withdrawal, suggesting that the relevance of oral frailty may extend beyond physical health alone [20,21,22]. Intervention studies further suggest that oral function may be modifiable, supporting the importance of early identification and community-based management [23]. These findings indicate that oral frailty is becoming an important topic at the intersection of geriatric medicine, oral health, and public health.

First, most studies have examined oral frailty in relation to physical frailty, sarcopenia, disability, malnutrition, dietary variety, or oral-health-specific outcomes, whereas fewer have placed oral frailty within a broader multidimensional health framework that integrates physical, mental, and social health [24,25]. Second, the social dimension of health remains underexplored, although oral function may influence eating with others, speech, appearance-related confidence, willingness to go out, and social participation. Third, although Japan has led the conceptualization and cohort validation of oral frailty, evidence from other rapidly aging societies, especially large middle-income countries with substantial community-based care needs, remains limited [8]. Existing Chinese studies have reported a relatively high prevalence of oral frailty among community-dwelling older adults, with estimates ranging approximately from 30% to 60%; a recent meta-analysis estimated a pooled prevalence of 53% (95% CI: 42–65%) [24,26,27]. Studies among hospitalized older adults have similarly indicated a high burden of oral frailty and insufficient screening coverage [28]. However, most Chinese studies have primarily focused on prevalence estimates, associated factors, or selected physical outcomes, with limited attention to standardized multidimensional health outcomes [29,30].

Contemporary healthy aging frameworks emphasize the integrated assessment of physical, mental, and social health rather than isolated disease or functional indicators [2,5]. In China, the health industry standard Health Assessment for Older Adults (WS/T 802–2022) provides a national framework for evaluating multidimensional health among older adults [31,32]. This standard incorporates physical, mental, and social domains and generates an overall health classification, thereby offering a structured tool for community-based screening and health management. Although developed for the Chinese context, WS/T 802–2022 operationalizes a multidimensional concept of healthy aging that is broadly consistent with international principles of geriatric assessment [32]. From the perspective of oral–systemic health, oral function may be linked to general health through impaired dietary intake, chronic low-grade inflammation, reduced communication ability, and restricted social participation [33,34]. To our knowledge, empirical studies applying WS/T 802–2022 or comparable standardized multidimensional health frameworks to examine oral frailty in relation to physical, mental, social, and overall health remain limited.

To address these gaps, we conducted a community-based cross-sectional study among older adults in Chenzhou, Hunan Province, China. The primary objective was to examine the association between OF-8-defined oral frailty and multidimensional health status based on the WS/T 802–2022 framework. Secondary objectives were to estimate the prevalence of oral frailty, evaluate the consistency of findings using SOFT-6 as an alternative oral frailty definition and additional sensitivity analyses, and explore dose–response relationships between OF-8 total score and continuous health scores. We hypothesized that oral frailty would not only be associated with poorer physical health, but also with poorer overall and social health, supporting its potential relevance as a simple indicator of multidimensional health vulnerability in community-dwelling older adults.

## 2. Materials and Methods

### 2.1. Study Design, Setting, and Participants

This community-based cross-sectional study was conducted in July 2025 in Beihu District, Chenzhou, Hunan Province, China. The investigation was jointly implemented by the research team and local community health service institutions. Community-dwelling older adults aged 60 years or above were recruited through community-based health service activities and local community organizations in Beihu District. Recruitment was community-based and convenience-oriented rather than consecutive enrollment or population-based random sampling. Eligible participants were invited to participate in face-to-face questionnaire interviews, physical examinations, and oral health assessments.

Participants were eligible if they were aged 60 years or older, had resided in the study communities for at least six months, were able to complete the questionnaire and physical examinations, and provided written informed consent. Individuals were excluded if they had severe cognitive impairment, severe psychiatric disorders, aphasia, acute severe illness, recent major oral surgery within the previous three months, or were receiving radiotherapy or chemotherapy for malignant tumors.

A total of 498 older adults were initially recruited. After excluding 44 individuals, including 9 who withdrew during the questionnaire survey, 32 who did not complete the WS/T 802–2022 assessment, and 3 with missing WS/T 802–2022 sub-items that precluded score calculation, 454 participants were included in the final analysis. The sample size was estimated assuming an oral frailty prevalence of approximately 50%, a two-sided confidence level of 95%, and a maximum allowable error of 5%. The minimum required sample size was calculated as 384 participants. Considering an estimated 15% rate of invalid questionnaires or incomplete data, the target sample size was increased to 442. The final analytical sample satisfied this requirement. Details of the sample size estimation are provided in Supplementary Materials S1.1.

This study was reported in accordance with the Strengthening the Reporting of Observational Studies in Epidemiology (STROBE) guideline for cross-sectional studies. The STROBE checklist is provided in Supplementary Materials S4.

### 2.2. Assessment of Oral Frailty

Oral frailty was assessed using the OF-8, which served as the primary exposure variable [10]. The OF-8 consists of eight items evaluating swallowing difficulty, chewing difficulty, oral dryness, number of remaining natural teeth, decreased biting force, repeated choking or coughing, oral motor function, and subjective chewing difficulty. Total scores range from 0 to 11, with higher scores indicating greater oral frailty burden. Participants with OF-8 scores of 4 or higher were classified as having oral frailty [10].

SOFT-6 was additionally used as an alternative oral frailty definition in sensitivity analyses [11]. SOFT-6 includes six items assessing swallowing difficulty, chewing difficulty, oral dryness, remaining teeth, repeated choking or coughing, and subjective chewing difficulty. Participants with SOFT-6 scores of 2 or higher were considered positive for oral frailty screening [11].

Both OF-8 and SOFT-6 assessments were administered by trained investigators using standardized face-to-face interviews and field assessment procedures. Items were scored according to the prespecified scoring protocol. Objectively verifiable items, including the number of remaining natural teeth, were confirmed through on-site oral examination. Details of scale items, scoring methods, and field assessment procedures are provided in Supplementary Materials S1.3.

### 2.3. Assessment of Multidimensional Health

Multidimensional health status was evaluated according to the Chinese health industry standard WS/T 802–2022 [31,32]. This framework evaluates overall health as well as physical, mental, and social health domains.

Overall health, physical health, mental health, and social health were categorized into three ordered levels: unhealthy, basically healthy, and healthy. The category “basically healthy” corresponds to an intermediate health status in the WS/T 802–2022 framework. These ordered categorical variables were used as outcomes in the primary ordinal regression analyses. Continuous scores for overall health and each health domain were additionally used in dose–response analyses, with higher scores indicating better health status.

The physical health domain includes indicators related to chronic disease management, activities of daily living, mobility and nutritional status. The mental health domain includes depression- and anxiety-related screening indicators, whereas the social health domain includes social participation, family and social support, and loneliness-related indicators. Details of the WS/T 802–2022 health classification are provided in Supplementary Materials S1.4.

### 2.4. Covariates and Additional Measurements

Covariates were selected based on previous literature and their potential associations with both oral frailty and multidimensional health. They included sociodemographic characteristics, lifestyle factors, chronic disease burden, anthropometric indicators, body composition measures, and dietary variables. Covariate selection was further guided by a directed acyclic graph. Age, sex, education, household income, chronic disease burden, smoking status, and alcohol consumption were considered potential confounders because they may be associated with both oral frailty and multidimensional health. BMI and weekly physical activity were included in the primary adjusted model but were interpreted cautiously because they may reflect both baseline health/lifestyle differences and pathway-related processes linking oral frailty with health status. Dietary variables were considered exploratory pathway-related variables rather than primary confounders. The directed acyclic graph and the rationale for covariate roles are provided in Supplementary Figure S1.

Sociodemographic characteristics included age, sex, education level, household income, marital status, and living arrangement. Lifestyle factors included smoking status, alcohol consumption, and weekly physical activity. Chronic disease burden was defined as the number of self-reported chronic conditions, including hypertension, diabetes, coronary heart disease, and hyperlipidemia. Detailed operational definitions and coding of covariates are provided in Supplementary Materials S1.7.

Physical examinations included measurements of height, weight, blood pressure, resting heart rate, grip strength, and body composition. Body mass index was calculated as weight in kilograms divided by height in meters squared. Body composition was assessed using a multifrequency bioelectrical impedance analyzer after participants had rested and removed metallic accessories. Appendicular skeletal muscle mass was used to calculate skeletal muscle mass index as appendicular skeletal muscle mass divided by height squared. Grip strength was measured using a standardized electronic handgrip dynamometer, and the maximum value obtained from repeated measurements was used for analysis. Details of body composition measurement are provided in Supplementary Materials S1.5.

Dietary intake was assessed using a food frequency questionnaire combined with a 24 h dietary recall [35]. The 24 h dietary recall was conducted by trained investigators through face-to-face interviews. Participants were asked to report all foods, beverages, cooking oils, condiments, and drinking water consumed during the preceding 24 h. Portion sizes were estimated using standard household measures and food photographs or portion-size aids when available. Nutrient intake was estimated according to the Chinese Food Composition Tables [36]. Dietary variables were used in descriptive analyses, covariate adjustment, and supplementary exploratory analyses. Details of dietary assessment and dietary index calculations are provided in Supplementary Materials S1.6. Because dietary intake may partly lie on the causal pathway between oral frailty and health, the two 24-h-recall-derived dietary variables (E-DII13 and 24 h dietary diversity) were added only in Model 4 as an exploratory dietary-adjusted model.

### 2.5. Statistical Analysis

Baseline characteristics were summarized according to OF-8-defined oral frailty status. Continuous variables were expressed as mean ± standard deviation or median with interquartile range, as appropriate. Categorical variables were presented as frequencies and percentages. Group comparisons were conducted using Student’s *t*-test, Wilcoxon rank-sum test, chi-square test, or Fisher’s exact test, as appropriate. Standardized mean differences were calculated to evaluate covariate imbalance, with values greater than 0.10 indicating meaningful imbalance.

The primary analyses examined associations between OF-8-defined oral frailty and ordered multidimensional health outcomes using cumulative-link (proportional odds) ordinal logistic regression models. Oral frailty was modeled as a binary exposure (present vs. absent, with absence as the reference category); the reported odds ratios therefore contrast participants with oral frailty against those without oral frailty, rather than representing the effect of a one-unit change in the OF-8 score. In the cumulative-link model, the odds ratio expresses the odds of being in a better (higher) health category versus all lower categories combined, and this odds ratio is assumed constant across the ordered category thresholds (the proportional odds assumption). Accordingly, an odds ratio less than 1 indicates that participants with oral frailty had lower odds of being in a better health category—that is, poorer multidimensional health—than participants without oral frailty. For the physical health outcome, which violated the proportional odds assumption, partial proportional odds models were used, and two threshold-specific odds ratios were reported (one for being at least basically healthy versus unhealthy and one for being healthy versus at most basically healthy); each threshold-specific odds ratio carries the same “with vs. without oral frailty” interpretation at its respective cut-point. Results were reported as odds ratios with 95% confidence intervals.

Four progressively adjusted models were constructed. Model 1 was adjusted for age and sex. Model 2 was additionally adjusted for socioeconomic variables, including education level and household income. Model 3 was further adjusted for body mass index, number of chronic diseases, smoking status, alcohol consumption, and weekly physical activity and was considered the primary adjusted model. Model 4 was additionally adjusted for two dietary variables derived from the 24 h dietary recall: E-DII13 and 24 h dietary diversity. This model was regarded as an exploratory dietary-adjusted model rather than the primary confounder-adjusted model because dietary intake may partly lie on the pathway linking oral frailty with multidimensional health.

The proportional odds assumption was evaluated using the Brant test [37]. For outcomes satisfying this assumption, cumulative-link ordinal logistic regression was used as the main model. For outcomes violating the proportional odds assumption, partial proportional odds models were used as the principal alternative model for interpretation, with conventional ordinal logistic regression estimates retained for comparison. Multinomial logistic regression models were also performed as supplementary analyses to assess whether associations were consistent across specific health-category comparisons. Linear regression analyses using continuous health scores were conducted as additional supplementary analyses. Details of the statistical models are provided in Supplementary Materials S1.8.

Several sensitivity analyses were performed to evaluate the robustness of the findings. First, SOFT-6 was used as an alternative oral frailty definition. Second, inverse probability weighting was used as a covariate-balancing sensitivity analysis to improve comparability between participants with and without oral frailty. Propensity scores were estimated using a generalized boosting model, stabilized weights were generated, and covariate balance before and after weighting was assessed using standardized mean differences [38]. Third, E-values were calculated to estimate the minimum magnitude of unmeasured confounding that would be required to move the observed association to the null [39]. Fourth, resting heart rate was analyzed as an exploratory negative control outcome. Fifth, participants with extreme total energy intake, defined as less than 800 kcal/day or greater than 4200 kcal/day, were excluded in an additional sensitivity analysis.

Restricted cubic spline models were used to explore dose–response relationships between oral frailty scores and continuous multidimensional health scores [40]. Continuous health scores were used only for dose–response visualization and supplementary analyses. Four knots were placed at the 5th, 35th, 65th, and 95th percentiles of the oral frailty score distribution. Models were adjusted according to Model 3. Overall association and nonlinearity *p* values were reported.

Participants with missing primary exposure or primary outcome data were excluded before the final analysis. Missing covariate data were handled using multiple imputation by chained equations under the missing-at-random assumption. Thirty imputed datasets were generated, and pooled estimates were calculated according to Rubin’s rules [41]. Details regarding missing data patterns and variables included in the imputation procedure are provided in the Supplementary Materials.

Exploratory mediation and risk stratification analyses were conducted only as supplementary analyses and were interpreted as hypothesis-generating because of the cross-sectional design. These analyses were not used to support causal inference or predictive claims in the primary manuscript.

All statistical tests were two-sided, and *p* values less than 0.05 were considered statistically significant. Overall health was prespecified as the primary outcome, whereas physical, mental, and social health domains were considered secondary outcomes. Therefore, results for domain-specific health outcomes were interpreted as secondary evidence. All analyses were performed using R software (R 4.5.2).

### 2.6. Ethics Statement

This study was conducted in accordance with the Declaration of Helsinki and was approved by the Biomedical Ethics Committee of Peking University (approval number: IRB00001052-25048). Written informed consent was obtained from all participants before enrollment. To ensure confidentiality, each participant was assigned a unique anonymous identification code, and all data were anonymized before analysis.

## 3. Results

### 3.1. Participant Characteristics

**Figure 1:**
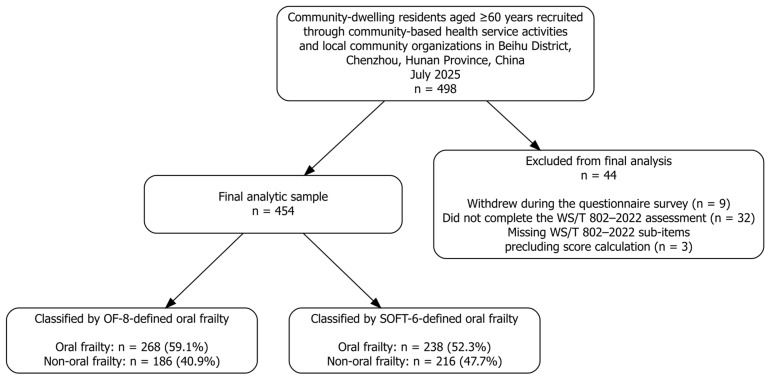
Flowchart of participant recruitment and inclusion. Note: A total of 498 community-dwelling residents aged 60 years or older were recruited and provided written informed consent in July 2025 in Beihu District, Chenzhou, China. Of these, 44 participants were excluded from the final analysis, including 9 who withdrew during the questionnaire survey, 32 who did not complete the WS/T 802–2022 assessment, and 3 who had missing WS/T 802–2022 sub-items precluding score calculation. The final analytic sample comprised 454 participants.

A total of 454 community-dwelling older adults were included in the final analysis (Figure 1). The mean age was 70.82 ± 7.48 years, and 310 participants were women (68.3%). Based on the OF-8 definition, 268 participants were classified as having oral frailty, corresponding to a prevalence of 59.1%. Based on SOFT-6, 238 participants were classified as screening positive for oral frailty, corresponding to a prevalence of 52.3%.

Baseline characteristics according to OF-8-defined oral frailty status are shown in Table 1. Compared with participants without oral frailty, those with oral frailty were older and had lower body mass index, lower skeletal muscle mass index, lower grip strength, greater chronic disease burden, and fewer remaining natural teeth. They also had lower overall health scores and physical health scores. Differences in mental and social health scores were smaller and did not reach statistical significance in the unadjusted comparisons. Extended baseline characteristics are provided in Supplementary Table S1, and baseline characteristics according to SOFT-6-defined oral frailty status are presented in Supplementary Table S2.

### 3.2. Association Between OF-8-Defined Oral Frailty and Multidimensional Health

**Figure 2:**
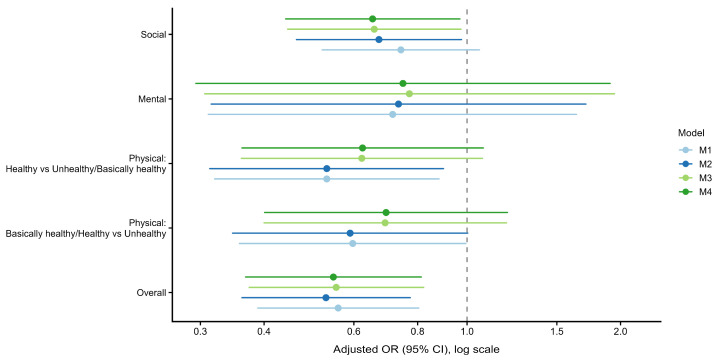
Adjusted associations between OF-8-defined oral frailty and multidimensional health. Note: M1: age, sex; M2: socioeconomic status; M3: BMI, number of chronic diseases, lifestyle; M4: dietary habits. The overall/mental/social variables were derived from a cumulative-link ordinal model; the physical variables, due to violations of the proportional odds assumption, were presented as two threshold-specific odds ratios (ORs) for PPO. The dashed line represents OR = 1.

The proportional odds assumption was assessed using the Brant test. The assumption was generally satisfied for overall, mental, and social health outcomes, but was violated for the physical health outcome. Therefore, partial proportional odds models were used as the principal models for the interpretation of physical health, and multinomial logistic regression models were used as supplementary analyses to examine consistency across health-category comparisons. The results are shown in Figure 2 and Table 2.

In the main adjusted model (Model 3), OF-8-defined oral frailty was associated with lower odds of being in a better overall health category (OR = 0.554, 95% CI: 0.374–0.822, *p* = 0.003). Because the proportional odds assumption was violated for physical health, this domain was interpreted using threshold-specific estimates from partial proportional odds models. In Model 3, OF-8-defined oral frailty showed directionally lower odds of better physical health at both thresholds, but neither threshold-specific association reached statistical significance. The OR was 0.669 (95% CI: 0.396–1.127, *p* = 0.131) for being basically healthy or healthy versus unhealthy and 0.595 (95% CI: 0.354–1.000, *p* = 0.050) for being healthy versus unhealthy or basically healthy. Therefore, the physical-health finding was interpreted as secondary and less definitive. Additional adjustment for dietary variables in Model 4 yielded similar estimates. OF-8-defined oral frailty remained associated with poorer overall health (OR = 0.547, 95% CI: 0.368–0.813, *p* = 0.003). For physical health, the threshold-specific PPO estimates remained directionally inverse but non-significant: OR = 0.671 (95% CI: 0.398–1.132, *p* = 0.135) for basically healthy or healthy versus unhealthy, and OR = 0.597 (95% CI: 0.355–1.004, *p* = 0.052) for healthy versus unhealthy or basically healthy. The association with social health was nominal, whereas the association with mental health remained non-significant.

In supplementary multinomial logistic regression analyses, using the unhealthy category as the reference, the associations between OF-8-defined oral frailty and basically healthy or healthy categories were generally consistent in direction with the ordinal regression results. These findings suggest that the main conclusions were not materially driven by the proportional odds assumption. Detailed results are provided in Supplementary Table S5.

### 3.3. Sensitivity Analyses

Sensitivity analyses are summarized in Table 3. When SOFT-6 was used as an alternative definition of oral frailty, the direction of associations was broadly consistent with the primary OF-8 analysis. In Model 3, SOFT-6-defined oral frailty was associated with lower odds of being in a better overall health category (OR = 0.564, 95% CI: 0.384–0.828, *p* = 0.003). For physical health, the conventional ordinal estimate was inverse (OR = 0.441, 95% CI: 0.250–0.779, *p* = 0.005); however, because the proportional odds assumption was violated for this outcome, this pooled estimate was reported only for cross-method comparison and was not interpreted as a primary threshold-specific result. SOFT-6-defined oral frailty was also associated with poorer mental health (OR = 0.197, 95% CI: 0.061–0.635, *p* = 0.007), whereas the association with social health was not statistically significant (OR = 0.841, 95% CI: 0.579–1.223, *p* = 0.365). Full SOFT-6 models are provided in Supplementary Tables S6 and S7.

In the inverse probability weighting analysis, covariate balance improved after weighting, with all weighted standardized mean differences below 0.10. The IPTW-weighted conventional ordinal models yielded inverse estimates for overall health, physical health, and social health. For physical health, the weighted pooled estimate was interpreted only as a supplementary directional comparison and did not replace the threshold-specific PPO estimates in Table 2. OF-8-defined oral frailty was associated with poorer overall health (weighted OR = 0.554, 95% CI: 0.382–0.802, *p* = 0.002), physical health (weighted OR = 0.571, 95% CI: 0.345–0.944, *p* = 0.029), and social health (weighted OR = 0.672, 95% CI: 0.468–0.965, *p* = 0.031). The association with mental health remained non-significant (weighted OR = 0.862, 95% CI: 0.381–1.950, *p* = 0.721). Covariate balance before and after weighting is shown in Supplementary Figure S5.

E-value analyses suggested that the associations with overall and physical health would require unmeasured confounding of modest-to-moderate strength to be fully explained. The E-values for the point estimates were 2.01 for overall health and 2.20 for physical health, with corresponding E-values for the confidence interval limits of 1.43 and 1.29, respectively. The E-values for mental and social health were smaller, indicating greater sensitivity to potential unmeasured confounding. Detailed E-value results are provided in Supplementary Table S9.

In the exploratory negative control outcome analysis, OF-8-defined oral frailty was not significantly associated with resting heart rate after adjustment for Model 3 covariates (β = 0.220, SE = 0.136, *p* = 0.107), providing no clear evidence of a broad systematic false-positive association.

After excluding participants with extreme total energy intake, the association between OF-8-defined oral frailty and overall health remained statistically significant (OR = 0.629, 95% CI: 0.412–0.962, *p* = 0.033). Associations with physical health (OR = 0.579, 95% CI: 0.305–1.099, *p* = 0.095) and social health (OR = 0.669, 95% CI: 0.440–1.017, *p* = 0.060) were attenuated and no longer reached conventional statistical significance, while the association with mental health remained non-significant (OR = 0.754, 95% CI: 0.254–2.241, *p* = 0.611). Detailed results are provided in Supplementary Table S11.

### 3.4. Dose–Response Associations Between Oral Frailty Scores and Health Scores

**Figure 3:**
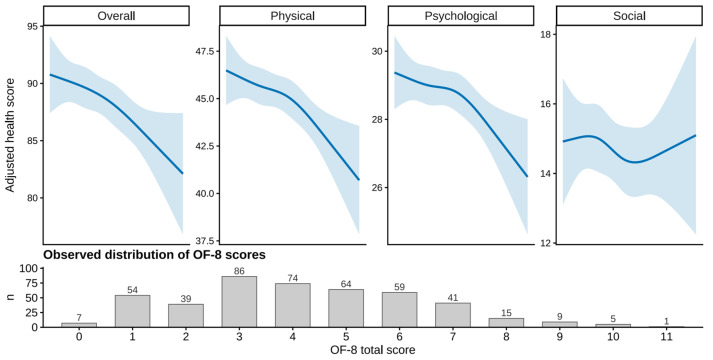
Dose–Response Associations Between OF-8 Score and Health Score.

Restricted cubic spline analyses were used to examine the associations between OF-8 total score and continuous multidimensional health scores (Figure 3). The OF-8 score showed significant inverse associations with overall health score (overall *p* = 0.002), physical health score (overall *p* < 0.001), and mental health score (overall *p* = 0.003). The association with social health score was not statistically significant (overall *p* = 0.588).

Tests for nonlinearity were not statistically significant for any of the four health outcomes, suggesting an approximately monotonic inverse association rather than a clear threshold or nonlinear pattern. In general, higher OF-8 scores were associated with lower overall, physical, and mental health scores. Dose–response analyses using SOFT-6 showed similar inverse associations with overall, physical, and mental health scores and are provided in Supplementary Table S13 and Supplementary Figure S4.

### 3.5. Exploratory Supplementary Analyses

Exploratory mediation and risk stratification analyses are presented in the Supplementary Materials and should be interpreted as hypothesis-generating because of the cross-sectional design. No statistically significant indirect pathways through body composition indicators or dietary indices were identified. Adding OF-8 to a demographic model modestly increased apparent discrimination for poor health status, but the improvement was not statistically significant in DeLong testing and was not interpreted as evidence of a validated prediction model.

## 4. Discussion

In this community-based cross-sectional study of older adults in China, oral frailty was common and was most consistently associated with poorer overall health as assessed using the WS/T 802–2022 framework. Evidence for domain-specific health outcomes was weaker and should be interpreted as secondary and exploratory. For physical health, threshold-specific estimates from partial proportional odds models were directionally consistent but not statistically significant. The association with social health was nominal and was not consistently supported across sensitivity analyses, whereas the association with mental health was not statistically significant in the primary OF-8 analysis. Sensitivity analyses using SOFT-6 and inverse probability weighting generally supported the main findings, and dose–response analyses suggested approximately monotonic inverse associations between OF-8 score and several continuous health scores. These findings suggest that oral frailty may be a simple indicator of broader multidimensional health vulnerability in community-dwelling older adults, rather than merely a localized oral condition. This interpretation is supported by recent evidence suggesting that oral frailty is associated with a range of adverse health outcomes, including physical frailty, sarcopenia, falls, malnutrition, deteriorating nutritional status, and low dietary diversity [42,43,44].

The prevalence of oral frailty in this study was 59.1% based on OF-8 and 52.3% based on SOFT-6, indicating that oral functional decline was common in this community sample. These estimates are broadly consistent with previous Chinese studies reporting oral frailty prevalence ranging approximately from 30% to 60%, as well as a recent meta-analysis estimating a pooled prevalence of 53% among community-dwelling older adults [24,26,27]. Studies among hospitalized older adults have also reported a high burden of oral frailty and insufficient screening coverage [28]. The relatively high prevalence observed in the present study may reflect the age structure, chronic disease burden, oral health status, and local health service context of the study population. More importantly, these findings support the need to consider oral functional decline as a relevant component of community-based geriatric health assessment [45].

The association between oral frailty and physical health is consistent with international evidence linking oral frailty or oral hypofunction to physical frailty, functional disability, and mortality [17,18,19]. Oral function is closely related to chewing efficiency, swallowing safety, food selection, and nutritional adequacy, all of which may be relevant to physical function in later life [14]. Oral frailty may also share common aging-related pathways with sarcopenia, malnutrition, chronic low-grade inflammation, and other geriatric syndromes [15,16]. However, these explanations should be interpreted as hypotheses rather than causal pathways. Because this study was cross-sectional, it cannot determine whether oral frailty precedes poorer physical health, results from declining general health, or reflects shared vulnerability associated with aging and multimorbidity. The association between oral frailty and poorer multidimensional health may also be partly understood through nutrition-related pathways. Older adults with oral frailty may avoid hard, fibrous, or protein-rich foods because of chewing difficulty, swallowing problems, oral dryness, reduced occlusal force, or tooth loss. Such changes may reduce dietary variety and compromise the intake of protein, energy, and micronutrients, thereby increasing nutritional vulnerability and contributing to declines in muscle mass, muscle strength, and physical resilience [34]. Previous studies have linked oral frailty or oral hypofunction with poorer nutritional status, lower dietary variety, and inadequate protein intake, while evidence from Chinese older adults also suggests that dietary diversity may mediate the association between tooth loss and frailty [35,36]. In the present study, additional adjustment for dietary inflammation index and 24 h dietary diversity yielded similar estimates, suggesting that the observed associations were not fully explained by the dietary indicators measured in this study. However, this finding should not be interpreted as evidence against nutritional mechanisms, because dietary intake was assessed at a single time point and may not fully reflect long-term dietary quality, nutritional reserves, or cumulative nutrition-related vulnerability.

In contrast to our initial hypothesis, the evidence linking oral frailty with social health was less consistent than that observed for overall health. Although OF-8-defined oral frailty showed a nominal association with poorer social health in the conventional adjusted ordinal model, this finding was not consistently supported across alternative oral-frailty definitions and sensitivity analyses. Therefore, the present results should not be interpreted as demonstrating a robust independent association between oral frailty and social health. A plausible relationship between oral function and social participation remains biologically and behaviorally reasonable because chewing difficulty, swallowing problems, tooth loss, oral dryness, or reduced confidence in eating and speaking may influence eating with others, communication, willingness to go out, and interpersonal engagement. However, our findings suggest that this pathway was not clearly captured by the WS/T 802–2022 social health classification in the present sample. The relatively crude three-level social health outcome, limited sample size in some categories, and cross-sectional design may have reduced our ability to detect domain-specific associations. Future longitudinal studies using more detailed measures of social participation, social isolation, and oral-health-related quality of life are needed to clarify whether oral frailty contributes to subsequent deterioration in social health.

Compared with physical outcomes, the social dimension of oral frailty has been less frequently investigated. Recent work has increasingly emphasized that oral function may affect well-being and social engagement through nutritional, mental, and social pathways [46]. Oral function may influence social health through difficulty eating with others, impaired speech, reduced appearance-related confidence, reluctance to go out, and decreased social participation [47]. This interpretation is consistent with previous research suggesting that oral hypofunction may be associated with social withdrawal among older adults [20]. The present study extends this perspective by examining social health within a standardized multidimensional healthy aging framework. Although the present social-health findings were not robust, they indicate that social pathways remain a plausible area for future research rather than a confirmed conclusion of this study.

The findings for mental health were less consistent. OF-8-defined oral frailty showed a directionally inverse but non-significant association with mental health, whereas SOFT-6-defined oral frailty was significantly associated with poorer mental health. One possible explanation is that SOFT-6 includes more subjective symptom-based items and may therefore be more closely related to perceived oral difficulties, mental distress, or self-rated health. In contrast, OF-8 captures a broader oral functional burden that may be more strongly aligned with physical and overall health. This instrument-specific finding should be interpreted cautiously and regarded as hypothesis-generating. Future studies should compare the performance of different oral frailty tools in relation to mental and social health outcomes.

The dose–response findings further support the relevance of oral frailty burden. Restricted cubic spline analyses showed approximately monotonic inverse associations between OF-8 score and overall, physical, and mental health scores, without evidence of a clear nonlinear threshold. This suggests that the health relevance of oral frailty may not be limited to a dichotomous screening cut-off. The continuous OF-8 score may reflect the cumulative burden of oral functional decline beyond the dichotomous oral frailty threshold. However, this cross-sectional study did not evaluate diagnostic accuracy, predictive performance, or clinically useful cut-points for multidimensional health outcomes. Any potential use of OF-8 to guide further multidimensional assessment therefore requires prospective validation.

Several sensitivity analyses provided supportive, although not definitive, evidence for the consistency of the main findings. The use of SOFT-6 as an alternative oral frailty definition and inverse probability weighting yielded broadly consistent associations with overall and physical health. E-value analyses suggested that the associations with overall and physical health would require unmeasured confounding of modest-to-moderate strength to be fully explained, although the confidence-limit E-values were relatively small. The exploratory negative control outcome analysis using resting heart rate did not suggest a clear systematic false-positive association, but resting heart rate may not represent a perfect negative control outcome. After excluding participants with extreme energy intake, the association with overall health remained significant, whereas associations with physical and social health were attenuated. Taken together, these analyses support the overall consistency of the findings but do not eliminate the possibility of residual confounding or measurement-related uncertainty.

Exploratory mediation analyses did not identify statistically significant indirect pathways through body composition indicators or dietary indices. These null findings should not be interpreted as evidence against nutritional or muscle-related mechanisms. Exposure, potential mediators, and outcomes were measured at the same time, making it impossible to establish temporal ordering. In addition, single-time-point dietary assessment and body composition measurement may not adequately capture long-term nutritional and functional trajectories. Therefore, these supplementary analyses should be regarded as hypothesis-generating and should not be used to support causal mediation. Future longitudinal studies should include repeated dietary assessments, validated malnutrition screening tools, objective nutritional biomarkers, and detailed measures of protein intake and dietary quality to clarify whether nutritional deterioration mediates the relationship between oral frailty and multidimensional health. Intervention studies combining oral function improvement with nutrition support may also help determine whether oral frailty is a modifiable entry point for healthy aging strategies.

This study has several strengths. First, it used the WS/T 802–2022 framework to evaluate overall, physical, mental, and social health, thereby placing oral frailty within a multidimensional healthy aging context. Second, oral frailty was assessed using OF-8 as the primary measure and SOFT-6 as an alternative definition, allowing the consistency of findings across screening tools to be examined. Third, the statistical analyses accounted for the ordered nature of health outcomes and evaluated the proportional odds assumption. Fourth, multiple sensitivity analyses were performed, including inverse probability weighting, E-value analysis, negative control outcome analysis, and exclusion of participants with extreme energy intake.

Several limitations should also be acknowledged. First, the cross-sectional design precludes causal inference and does not allow determination of the temporal relationship between oral frailty and multidimensional health. Second, participants were recruited from one urban district in Hunan Province, which may limit generalizability to rural populations, other regions of China, or older adults in different health care systems. Community-based recruitment may also have introduced selection bias, as older adults who were willing and able to participate in field assessments may differ from those who did not participate. The number of older adults approached and the number of refusals were not systematically recorded, which further limits assessment of selection bias. Third, although multiple covariates were adjusted for, residual confounding from unmeasured factors such as oral hygiene behaviors, dental service use, medication use, inflammatory status, and the broader socioeconomic context cannot be ruled out. Fourth, some variables, including lifestyle behaviors, chronic disease history, dietary intake, and components of oral frailty and mental or social health assessment, were based on self-report and may be affected by recall bias or common method bias. Finally, exploratory mediation and risk stratification analyses were supplementary and should not be interpreted as evidence of causal pathways or a validated prediction model. In addition, the available dietary indicators, including 24 h dietary diversity and dietary inflammation index, may not fully capture habitual dietary patterns, protein quality, micronutrient adequacy, or long-term nutritional status. Furthermore, a limited conceptual overlap exists between certain nutrition/eating-related indicators (MNA-SF food intake items and eating status) in the Physical Health domain of WS/T 802–2022 and the oral frailty item, which may introduce potential criterion contamination into the association between physical health and (indirectly) overall health; refer to Supplementary S1.4 for indicator-level comparisons.

## 5. Conclusions

In this community-based cross-sectional study of older adults in China, oral frailty was common and was most consistently associated with poorer overall health as assessed using the WS/T 802–2022 framework. Evidence for physical, mental, and social health domains was less consistent and should be interpreted as secondary and exploratory. Threshold-specific physical-health estimates were directionally consistent but not statistically significant, and the social-health association was not robust across sensitivity analyses. Because the study was cross-sectional and because limited conceptual overlap exists between oral frailty items and nutrition/eating-related indicators in WS/T 802–2022, causal interpretation is not warranted. These findings suggest that oral frailty may be a simple, nutrition-relevant indicator that could help flag older adults for fuller multidimensional geriatric assessment. However, because the study was cross-sectional and associations beyond overall health were not consistent, whether oral frailty has practical value as a screening or triage indicator was not formally evaluated and remains to be established in longitudinal, multicenter studies.

## Figures and Tables

**Table 1:** Baseline Characteristics of Participants According to OF-8-Defined Oral Frailty Status.

Variables	Total (*n* = 454)	No Oral Frailty (*n* = 186)	Oral Frailty (*n* = 268)	*p*	SMD
Age (year, mean ± SD)	70.82 ± 7.48	69.19 ± 7.08	71.95 ± 7.55	<0.001	0.376
Sex-female (%)	310 (68.3)	128 (68.8)	182 (67.9)	0.919	0.019
Education-secondary or higher (%)	290 (64.1)	116 (62.4)	174 (64.9)	0.440	0.156
BMI (kg/m^2^, mean ± SD)	22.82 ± 3.42	23.35 ± 3.41	22.44 ± 3.39	0.006	0.266
SMI (kg/m^2^, mean ± SD)	15.88 ± 1.74	16.08 ± 1.80	15.73 ± 1.69	0.034	0.204
Grip (kg, mean ± SD)	22.29 ± 11.80	23.72 ± 12.71	21.32 ± 11.06	0.045	0.201
DBP (mmHg, mean ± SD)	79.15 ± 10.67	81.16 ± 11.25	77.81 ± 10.06	0.002	0.314
SBP (mmHg, mean ± SD)	132.90 ± 19.47	133.29 ± 20.16	132.64 ± 19.03	0.742	0.033
HR (beats/min, mean ± SD)	79.99 ± 11.64	80.52 ± 11.07	79.64 ± 12.02	0.457	0.076
Diabetes (%)	68 (15.0)	19 (10.2)	49 (18.3)	0.025	0.232
Coronary heart disease (%)	89 (19.6)	25 (13.4)	64 (23.9)	0.008	0.270
Hyperlipidemia (%)	128 (28.2)	41 (22.0)	87 (32.5)	0.020	0.236
Number of chronic diseases (mean ± SD)	1.02 ± 1.05	0.83 ± 0.97	1.16 ± 1.07	0.001	0.325
Number of remaining natural teeth (mean ± SD)	22.09 ± 8.80	23.83 ± 7.87	20.94 ± 9.21	0.001	0.337
Overall health score (mean ± SD)	85.66 ± 9.74	87.41 ± 8.89	84.44 ± 10.13	0.001	0.312
Physical health scores (mean ± SD)	44.26 ± 5.37	45.48 ± 4.55	43.40 ± 5.73	<0.001	0.402
Mental health scores (mean ± SD)	27.98 ± 2.93	28.24 ± 2.30	27.80 ± 3.29	0.118	0.154
Social health scores (mean ± SD)	13.43 ± 4.54	13.69 ± 4.86	13.24 ± 4.30	0.294	0.099

Note: SD = standard deviation; BMI = body mass index; SMI = skeletal muscle mass index; DBP = diastolic blood pressure; SMD = standardized mean difference. Continuous variables were compared using the *t*-test or the Wilcoxon rank-sum test, while categorical variables were analyzed using the χ^2^ test.

**Table 2:** Association Between OF-8-Defined Oral Frailty and Multidimensional Health.

Outcome	Model	Contrast	OR	95% CI	*p*	FDR_*P*
Overall health N (U/B/H): 90/217/147	M1		0.559	0.389–0.804	0.002	0.010
	M2		0.529	0.362–0.774	0.001	0.005
	M3		0.554	0.374–0.822	0.003	0.015
	M4		0.547	0.368–0.813	0.003	0.015
Physical health N (U/B/H): 83/15/356	M1	≥Basically healthy vs. Unhealthy	0.597	0.358–0.994	0.047	0.078
	M1	Healthy vs. ≤Basically healthy	0.531	0.320–0.881	0.014	0.035
	M2	≥Basically healthy vs. Unhealthy	0.575	0.346–0.956	0.033	0.045
	M2	Healthy vs. ≤Basically healthy	0.512	0.309–0.848	0.009	0.023
	M3	≥Basically healthy vs. Unhealthy	0.669	0.396–1.127	0.131	0.164
	M3	Healthy vs. ≤Basically healthy	0.595	0.354–1.000	0.050	0.083
	M4	≥Basically healthy vs. Unhealthy	0.671	0.398–1.132	0.135	0.169
	M4	Healthy vs. ≤Basically healthy	0.597	0.355–1.004	0.052	0.087
Mental health N (U/B/H): 17/11/426	M1		0.715	0.311–1.639	0.428	0.428
	M2		0.734	0.315–1.710	0.473	0.473
	M3		0.771	0.306–1.946	0.582	0.582
	M4		0.749	0.294–1.908	0.544	0.544
Social health N (U/B/H): 119/164/171	M1		0.742	0.520–1.057	0.098	0.123
	M2		0.672	0.463–0.975	0.036	0.045
	M3		0.658	0.445–0.973	0.036	0.083
	M4		0.653	0.441–0.968	0.034	0.085

Note: Model M1 adjusted for age and sex; Model M2 further adjusted for education and income; Model M3 additionally adjusted for BMI, number of chronic diseases, smoking, alcohol consumption, and physical activity; and Model M4 further adjusted for dietary inflammation index and 24 h dietary diversity based on Model M3. For physical health, estimates are threshold-specific ORs from partial proportional odds models because the proportional odds assumption was violated. Other outcomes were analyzed using cumulative-link ordinal logistic regression models. All multiple-imputation analyses included 454 participants. FDR-adjusted *p* values were calculated using the Benjamini–Hochberg method within each model across five health-related tests: overall health, two threshold-specific physical-health contrasts, mental health, and social health. The number of participants in each health category is reported under each outcome label as N (U/B/H), corresponding to unhealthy (U), basically healthy (B), and healthy (H); the three counts sum to 454 for every outcome.

**Table 3:** Sensitivity analyses for the association between oral frailty and multidimensional health.

Outcome	Conventional OF-8 Ordinal Estimate OR (95% CI), *p*	SOFT-6 Alternative Definition OR (95% CI), *P*	IPTW-Weighted Model OR (95% CI), *p*	E-Value Point/CI Limit
Overall health	0.554 (0.374–0.822), *p* = 0.003	0.564 (0.384–0.828), *p* = 0.003	0.554 (0.382–0.802), *p* = 0.002	2.01/1.43
Physical health	0.489 (0.268–0.891), *p* = 0.020	0.441 (0.250–0.779), *p* = 0.005	0.571 (0.345–0.944), *p* = 0.029	2.20/1.29
Mental health	0.771 (0.306–1.946), *p* = 0.582	0.197 (0.061–0.635), *p* = 0.007	0.862 (0.381–1.950), *p* = 0.721	1.45/1.00
Social health	0.658 (0.445–0.973), *p* = 0.036	0.841 (0.579–1.223), *p* = 0.365	0.672 (0.468–0.965), *p* = 0.031	1.75/1.10

Notes: Values are ORs with 95% CIs. OR < 1 indicates lower odds of being in a better health category among participants with oral frailty. M3 adjusted for age, sex, education, income, BMI, chronic disease count, smoking, drinking, and physical activity. The IPTW-weighted estimates were based on stabilized inverse probability weights. E-values were calculated for the main adjusted associations to assess the minimum strength of unmeasured confounding required to explain away the observed associations. OF-8, Oral Frailty Index-8; SOFT-6, six-item screening tool for oral frailty; IPTW, inverse probability of treatment weighting. For physical health, the proportional odds assumption was violated; the pooled ordinal odds ratios shown here (including the SOFT-6 and IPTW columns) are presented only for cross-method comparison. The primary interpretation for physical health relies on the threshold-specific partial proportional odds estimates in Table 2, which were not statistically significant.

## Data Availability

The data presented in this study are available on request from the corresponding author due to ethical, privacy, and institutional restrictions. The data are not publicly available because they contain information derived from human participants. De-identified data may be made available upon reasonable request, subject to approval by the Biomedical Ethics Committee of Peking University and completion of an appropriate data use agreement.

## Supplementary Materials

The supporting information can be downloaded at: https://www.mdpi.com/article/10.3390/nu18142250/s1, References [17,36,39,48,49,50,51,52,53,54] are cited in the Supplementary Materials.
