# Supplementary material for: Oral Frailty and Multidimensional Health Among Community-Dwelling Older Adults in China: A Cross-Sectional Study
Source: Nutrients. 2026 Jul 9;18(14):2250. doi: 10.3390/nu18142250 (PMC13414565; doi:10.3390/nu18142250)
Supplement: Supplementary file 1 [file nutrients-18-02250-s001.zip › Supplementary Materials.pdf]

Supplementary Materials: Oral Frailty and Multidimensional Health among Community-Dwelling Older Adults in China: A Cross-Sectional Study

This supplementary material includes:

S1 Extended Methods (covering questionnaire items, scale scores, health classification, body composition and dietary assessment, definition of covariates, and additional statistical methods);

S2 Supplementary Tables S1–S23;

S3 Supplementary Figures S1–S14;

S4 STROBE Cross-sectional Study Report Checklist;

S5 Supplementary References.

S1 Expansion Methods

S1.1 Study Design and Sample Size Estimation

This study conducted a face-to-face cross-sectional survey in Beihu District, Chenzhou City, Hunan Province in July 2025. The sample size was estimated based on an expected oral frailty prevalence of approximately 50%, a maximum allowable absolute error of 5%, and a significance level of $\alpha = 0.05$, yielding $n \approx 1.96^2 \times 0.5 \times 0.5 / 0.05^2 = 384$; accounting for approximately 15% of invalid questionnaires and missing covariate data, the target sample size was set at 442. A total of 498 community residents aged ≥60 years were invited, all of whom provided informed consent; after excluding 44 participants with missing key variables or who did not meet inclusion criteria, the final analytical sample consisted of 454 cases.

S1.2 Inclusion and Exclusion Criteria

Inclusion criteria: (1) Age ≥60 years; (2) Residing in the study community for at least 6 months; (3) Adequate cognitive function to complete the questionnaire (basic information may be provided during a simple screening); (4) written informed consent. Exclusion criteria: (1) Severe mental disorders or aphasia; (2) Bedridden status preventing participation in physical measurements; (3) Within 3 months after major oral surgery; (4) Undergoing radiotherapy or chemotherapy for malignant tumors.

S1.3 Items and Scoring of the Oral Frailty Index (OF-8/SOFT-6)

The Oral Frailty Index-8 (OF-8) was developed by Tanaka et al. (2018) and consists of eight items that assess: (1) decline in swallowing function; (2) decline in mastication function; (3) dry mouth; (4) number of remaining natural teeth; (5) bite force; (6) recurrent choking; (7) tongue-lip motor function (oral alternation movement, ODM); and (8) subjective masticatory difficulty. Each item is scored on a 0–1 scale based on risk level (some items may range from 0 to 2), yielding a total score of 0–11, with a total score ≥4 defined as oral frailty. SOFT-6 comprises six items covering swallowing, mastication, dry mouth, remaining teeth, recurrent choking, and subjective masticatory difficulty, with a total score ≥2 indicating a positive oral frailty screening result. In this study, both the OF-8 and SOFT-6 assessments were conducted by trained investigators through on-site questioning and measurement; the number of remaining natural teeth was determined via clinical oral examination.

S1.4 WS/T 802–2022 "Health Assessment of the Elderly"

Multidimensional health was evaluated according to the Chinese health industry standard WS/T 802–2022, *The Standard for Healthy Chinese Older Adults*. The assessment consists of three domains with a total possible score of 100 points: physical health (0–50 points), mental health (0–30 points), and social health (0–20 points). The overall health score was calculated as the sum of the three domain scores. The physical health domain included general health status, activities of daily living, and disease status. General health status comprised nutritional status, sleep, vision, hearing, and eating status. Activities of daily living included basic activities of daily living and, for participants younger than 80 years, instrumental activities of daily living. Disease status included the control of health-related risk factors and chronic diseases. The mental health domain included cognitive function, anxiety, depression, life satisfaction, and health literacy. Health-literacy indicators included understanding of ageing, a balanced diet, regular and appropriate physical activity, smoking cessation and limited alcohol consumption, medication adherence, and regular health examinations. The social health domain included social participation, social adaptation, and social support. Social participation was assessed according to the frequency of participation in family and social activities; social adaptation was evaluated using the social-development and spiritual-cultural adaptation scale for older adults; and social support was assessed using the Lubben Social Network Scale.

Participants were classified as healthy, basically healthy, or unhealthy for each domain according to the prespecified scoring rules. These category thresholds were defined by WS/T 802–2022 and were not derived from the distribution of the present study sample. Potential conceptual overlap between oral frailty and the physical health assessment was limited mainly to nutrition- and eating-related indicators. In particular, the nutritional assessment included an item concerning reduced food intake due to appetite loss, digestive problems, or chewing or swallowing difficulties, and eating status was classified as a normal, semiliquid, or liquid diet. The WS/T 802–2022 assessment did not independently score the number of remaining teeth, oral dryness, biting force, oral motor function, or recurrent choking or coughing.

Table S0a Classification of physical, mental, social, and overall health according to WS/T 802–2022

Health outcome	Healthy	Basically healthy	Unhealthy
Physical health	Total score of 40–50 points, with no tertiary indicator scored 0	Total score of 30–39 points, with no tertiary indicator scored 0	Total score ≤29 points or any tertiary indicator scored 0
Mental health	Total score of 24–30 points, with no secondary indicator scored 0	Total score of 18–23 points, with no secondary indicator scored 0	Total score ≤17 points or any secondary indicator scored 0
Social health	Total score of 16–20 points	Total score of 12–15 points	Total score ≤11 points
Overall health	Total score of 80–100 points and all three health domains classified as healthy	Criteria for neither healthy nor unhealthy status were met	Total score ≤59 points, physical health classified as unhealthy, mental health classified as unhealthy, or social health score equal to 0

Note: *Physical, mental, and social health scores ranged from 0–50, 0–30, and 0–20 points, respectively. The overall health score was the sum of the three domain scores and ranged from 0 to 100. In multinomial logistic regression analyses, the unhealthy category of each corresponding outcome was used as the reference category.*

Table S0b Item-level mapping between WS/T 802–2022 health indicators and OF-8/SOFT-6 oral frailty items.

WS/T 802–2022 domain	Indicator/component	Operational content in WS/T 802–2022	Correspondence with OF-8/SOFT-6 items	Overlap judgement	Suggested interpretation
Physical health	Nutritional status	Mini Nutritional Assessment–Short Form (MNA-SF) and BMI-based nutritional classification.	Nutritional status is scored as 4 points for good nutrition (MNA-SF ≥12), 2 points for fair nutrition (MNA-SF 8–11 or 25 < BMI < 30), and 0 points for poor nutrition (MNA-SF <8 or BMI ≥30). Within the MNA-SF, the potentially overlapping sub-item asks whether food intake decreased during the past 3 months because of appetite loss, digestive problems, or chewing/swallowing difficulties. The sub-item is scored as 0 points for severe decrease in food intake, 1 point for moderate decrease, and 2 points for no decrease.	Indirect/conceptual overlap	This sub-item may partly reflect chewing or swallowing difficulty, which overlaps conceptually with OF-8/SOFT-6 swallowing and chewing items. However, it is embedded within a broader nutritional screening score and does not directly score remaining teeth, oral dryness, bite force, oral motor function, or recurrent choking/coughing.
Physical health	Sleep status	Presence and daily-life impact of sleep disturbance.	No OF-8/SOFT-6 item assesses sleep.	No direct overlap	Independent non-oral health indicator.
Physical health	Vision	Ability to read standard font with usual glasses if used.	No OF-8/SOFT-6 item assesses vision.	No direct overlap	Independent non-oral health indicator.
Physical health	Hearing	Ability to communicate and hear common sounds, with hearing aid if used.	No OF-8/SOFT-6 item assesses hearing.	No direct overlap	Independent non-oral health indicator.
Physical health	Eating status	Normal diet, semi-liquid diet, or liquid diet.	4 points: normal diet; 2 points: semi-liquid diet; 0 points: liquid diet.	Indirect/conceptual overlap	Eating status may be influenced by chewing or swallowing ability and therefore has potential conceptual overlap with oral frailty. However, it is a functional diet-form indicator and does not directly measure specific OF-8/SOFT-6 oral-function items.
Physical health	Basic activities of daily living	Basic self-care ability assessed according to the older-adult capacity evaluation standard.	No direct OF-8/SOFT-6 counterpart.	No direct overlap	General functional indicator rather than oral-function indicator.
Physical health	Instrumental activities of daily living	Telephone use, shopping, meal preparation, housework, transportation, medication use, and financial management for participants younger than 80 years.	No direct OF-8/SOFT-6 counterpart.	No direct overlap	General independent-living indicator.
Physical health	Health-related risk-factor control	Blood pressure, blood glucose, blood lipids,	No OF-8/SOFT-6 item assesses cardiometabolic risk-factor control.	No direct overlap	Independent clinical-risk indicator.

WS/T 802–2022 domain	Indicator/component	Operational content in WS/T 802–2022	Correspondence with OF-8/SOFT-6 items	Overlap judgement	Suggested interpretation
		and related risk-factor control.			
Physical health	Chronic disease status	Presence and control of chronic diseases and their impact on daily activities.	No OF-8/SOFT-6 item directly assesses chronic disease status.	No direct overlap	General disease-burden indicator.
Mental health	Cognitive function	Cognitive screening according to WS/T 484-related criteria.	No OF-8/SOFT-6 item directly assesses cognition.	No direct overlap	Distinct mental-health indicator.
Mental health	Anxiety	Generalized Anxiety Disorder-7 (GAD-7).	No OF-8/SOFT-6 item assesses anxiety.	No direct overlap	Distinct mental-health indicator.
Mental health	Depression	Geriatric Depression Scale-15 (GDS-15).	No OF-8/SOFT-6 item assesses depressive symptoms.	No direct overlap	Distinct mental-health indicator.
Mental health	Life satisfaction	Self-rated life satisfaction.	No OF-8/SOFT-6 item assesses life satisfaction.	No direct overlap	Distinct mental indicator.
Mental health	Health literacy	Understanding ageing, balanced diet, regular physical activity, smoking cessation/limited alcohol use, medication adherence, and regular health examinations.	Balanced diet has a weak diet-related conceptual link, but no direct oral-function item is scored.	Minimal conceptual overlap	Not an oral-frailty measure; can be described as lifestyle/health-literacy content.
Social health	Social participation	Frequency of participation in family and social activities during the past year.	No OF-8/SOFT-6 item directly measures social participation.	No direct item overlap	Could be a downstream consequence of oral problems but is not part of OF-8/SOFT-6 measurement.
Social health	Social adaptation	Older-adult social-development and spiritual-cultural adaptation scale.	No OF-8/SOFT-6 item measures social adaptation.	No direct overlap	Distinct social-health indicator.
Social health	Social support	Lubben Social Network Scale.	No OF-8/SOFT-6 item measures social support or social network size.	No direct overlap	Distinct social-health indicator.
Overall health	Overall WS/T 802–2022 classification	Sum of physical, mental, and social health scores, combined with decision rules for unhealthy status.	Inherits only the limited indirect overlap present in physical-health nutritional status and eating status.	Limited indirect overlap	Overall-health findings should be interpreted cautiously, but most components are not direct oral-frailty items.

Note: OF-8 includes swallowing difficulty, chewing difficulty, oral dryness, number of remaining natural teeth, decreased bite force, repeated choking/coughing, oral motor function, and subjective chewing difficulty. SOFT-6 includes swallowing difficulty, chewing difficulty, oral dryness, remaining teeth, repeated choking/coughing, and subjective chewing difficulty. WS/T 802–2022 does not directly score number of remaining teeth, oral dryness, decreased bite force, oral motor function, or repeated choking/coughing as health-domain outcome indicators.

S1.5 Body Composition Measurement

Body composition was measured by trained investigators using the InBody S10 multi-frequency bioelectrical impedance device (InBody, South Korea) after subjects had fasted, emptied their bladder, and rested for 5 minutes. Intense physical activity was prohibited for 4 hours prior to measurement, and metal accessories were removed during measurement. Skeletal muscle mass of the limbs was calculated as the sum of skeletal muscle masses (kg) divided by square height to obtain the skeletal muscle index (SMI; kg/m^2). Grip strength was measured using an EH101 electronic grip meter (CAMRY brand), with the maximum value among three repeated measurements taken from the dominant hand recorded (kg).

S1.6 Dietary Assessment and Index Calculation

Dietary intake was assessed using a food frequency questionnaire (FFQ) combined with a single 24-hour dietary recall. The FFQ was used to collect the habitual intake frequency of selected food groups, including vegetables, fruits, milk, and seafood. Intake frequency was recorded using predefined response categories and was used to describe habitual food-consumption patterns and construct selected frequency-based dietary variables.

The 24-hour dietary recall was conducted by trained investigators through face-to-face interviews using a standardized dietary-record form. Participants were asked to recall all foods and beverages consumed during the preceding 24 hours, including staple foods, dishes, snacks, beverages, cooking oils, salt, condiments, and drinking water. For each item, investigators recorded the food name, preparation method, estimated portion size, and amount consumed. Standard household measures, such as bowls, cups, spoons, and common portion-size references, were used to assist participants in estimating intake amounts. When mixed dishes were reported, ingredients were disaggregated as far as possible based on participant report and standard recipes. Foods consumed outside the home were also recorded when applicable.

Reported dietary items were converted into gram weights and linked to the Chinese Food Composition Tables to estimate total energy and nutrient intake. The 24-hour recall data were used to calculate total energy intake, nutrient intake, food-group intake, and 24-hour dietary diversity. Dietary indices derived from the 24-hour recall included the energy-adjusted dietary inflammatory index and 24-hour dietary diversity, which were used in the exploratory dietary-adjusted model. Other dietary indicators, such as nutrient adequacy and dietary balance indices, were used only in descriptive and exploratory supplementary analyses.

Because dietary variables may partly lie on the pathway between oral frailty and multidimensional health, the dietary-adjusted model was interpreted as exploratory rather than as the primary confounder-adjusted model. In addition, because the 24-hour dietary recall was conducted at a single time point, it may not fully capture long-term habitual dietary intake.

This study calculated and utilized the following four types of dietary indices:

(1) Energy-adjusted Dietary Inflammation Index (E-DII13)—calculated using the algorithm proposed by Shivappa et al. (2014), which identifies 13 inflammatory components obtainable from the 24-hour dietary recall data (including energy, fiber, ω-3 and ω-6 fatty acids, vitamin D, vitamin B12, magnesium, iron, zinc, selenium, etc.); a higher E-DII13 value indicates

stronger pro-inflammatory potential of the diet.

(2) Adequate nutrient ratio (MAR mean): Select nine key nutrients (protein, calcium, iron, zinc, selenium, vitamin A, vitamin C, B1, B2); divide the intake of each nutrient by its Recommended Nutrient Intake (RNI) and cap it at 1, then calculate the arithmetic mean within the range of 0–1.

(3) China Dietary Balance Index DBI-LBS — calculated based on the "China Dietary Guidelines 2022" to determine the low scores (LBS, Lower-Bound Score) for eight food categories, indicating the degree of deviation from adequate intake; a higher LBS value signifies more severe dietary insufficiency.

(4) China Healthy Dietary Index CHEI-9 (Simplified CHEI): It selects the nine scoring dimensions most relevant to this study from the CHEI scale (vegetables, fruits, whole grains, dairy products, soybeans, nuts, red/processed meat, sugary beverages, cooking oils), scores them according to CHEI standards, and sums the results, yielding a score ranging from 0 to 90.

In the main regression analyses, Model 4 additionally adjusted for two dietary variables derived from the 24-hour dietary recall: the energy-adjusted dietary inflammatory index based on 13 available dietary components (E-DII13) and 24-hour dietary diversity. These two variables were selected because they represent complementary aspects of dietary exposure: E-DII13 reflects the inflammatory potential of dietary intake, whereas 24-hour dietary diversity reflects the variety of foods consumed during the recall period.

Other dietary indicators, including the mean adequacy ratio (MAR), the Dietary Balance Index lower-bound score (DBI-LBS), and the simplified China Healthy Eating Index (CHEI-9), were calculated for descriptive and exploratory supplementary analyses only. They were not included in Model 4 to avoid overadjustment and collinearity among multiple dietary indices. Because dietary intake may partly lie on the pathway between oral frailty and multidimensional health, Model 4 was interpreted as an exploratory dietary-adjusted model rather than as the primary confounder-adjusted model.

S1.7 Definition of Covariates

Table S0c Operational Definitions of Covariates

Variable	Source	Operational Definition	Use in analysis
Age	Questionnaire	Continuous variable: year	Included in Model 1
Sex	Questionnaire	Male / Female	Included in Model 1
Per capita annual income of the family	Questionnaire	<30,000 yuan / 30,000–50,000 yuan / 50,000–70,000 yuan / 70,000–90,000 yuan / >90,000 yuan	Included in Model 2
Education	Questionnaire	Below elementary school; elementary school; middle school; high school; undergraduate level	Included in Model 2
BMI	Body Composition Analyzer	kg/m^2	Included in Model 3
Smoke	Questionnaire	Never/ Smoke-Free/In the past 30 days has / Frequently	Included in Model 3
Alcohol	Questionnaire	Never/ Alcohol-Free/In the past 30 days has / Frequently	Included in Model 3
SBP/DBP/HR	Electronic sphygmomanometer	Calculate the average value from two measurements taken using a blood pressure monitor.	Negative Control
Hypertension/diabetes / heart disease/hyperlipidemia	Questionnaire	Self-report of the presence of the aforementioned conditions	Included in Model 3
Number of Chronic Diseases	Questionnaire	Cumulative self-reported disease counts for hypertension, diabetes, coronary heart disease, and hyperlipidemia (0–4)	Included in Model 3
Frequency of exercise per week	Questionnaire	Never;1-2days/week;3-4days/week;5-6days/week;everyday	Included in Model 3
Selected food-group intake frequency	FFQ	Habitual intake frequency of selected food groups, including vegetables, fruits, milk, and seafood	Descriptive and supplementary exploratory analyses
Total energy intake	24-hour dietary recall	Total energy intake calculated from all foods and beverages consumed during the preceding 24 hours using the Chinese Food Composition Tables	Descriptive analyses and extreme-energy sensitivity analysis
Nutrient intake	24-hour dietary recall	Intake of macro- and micronutrients calculated from reported 24-hour food consumption using the Chinese Food Composition Tables	Used to derive dietary indicators
E-DII13	24-hour dietary recall	Energy-adjusted dietary inflammatory index calculated from 13 available dietary components	Included in Model 4
24-hour dietary diversity	24-hour dietary recall	Number or diversity score of food groups consumed during the 24-hour recall period	Included in Model 4
MAR	24-hour dietary recall	Mean adequacy ratio calculated from selected nutrients using recommended nutrient intake references	Exploratory supplementary analysis only
DBI-LBS	24-hour dietary recall / dietary guideline-based scoring	Lower-bound score of the Dietary Balance Index, reflecting dietary insufficiency	Exploratory supplementary analysis only

Variable	Source	Operational Definition	Use in analysis
CHEI-9	24-hour dietary recall / simplified dietary quality scoring	Simplified China Healthy Eating Index based on nine dietary components	Exploratory supplementary analysis only

S1.8 Additional Notes on Statistical Methods

The primary association analyses were conducted using cumulative-link ordinal logistic regression models. The proportional odds assumption was assessed for each ordinal outcome using the Brant test. For outcomes that violated this assumption, specifically physical health as shown in Table S3, a partial proportional odds (PPO) model was additionally fitted as a sensitivity analysis to allow threshold-specific effects. The overall conclusions were not materially altered. Missing covariate data were handled using multiple imputation by chained equations under the missing-at-random assumption. Thirty imputed datasets were generated. The imputation model included the primary exposure, multidimensional health outcomes, sociodemographic variables, lifestyle factors, chronic disease burden, anthropometric and body-composition indicators, and dietary variables. Variables used as predictors for each imputed variable are listed in Table. Pooled estimates were calculated according to Rubin’s rules. Complete-case analyses were conducted as sensitivity analyses and compared with the multiple-imputation results.

Table S0d Variables included in the multiple-imputation models

Variable role	Variables	Imputation method	Predictor variables included in the imputation model
Imputed continuous variables	BMI, SMI, body fat percentage, grip strength, tooth number, E-DII13, MAR, diet diversity, CHEI, etc.	Predictive mean matching	Age, sex, socioeconomic variables, lifestyle factors, chronic disease burden, oral frailty indicators, multidimensional health outcomes, body-composition variables, and dietary variables
Imputed binary variable	Probable sarcopenia	Logistic regression	Same predictor set as above
Variables included as predictors but not imputed	Age, sex, OF-8 status/score, SOFT-6 status/score, health-domain categories/scores, chronic disease count, etc.	Not applicable	Used as auxiliary predictors in the imputation model
Variables not included in the imputation model	Variables with no missingness or variables not used in primary models	Not applicable	Not applicable

Note: For each imputed variable, all variables listed in the predictor set were included as candidate predictors, except the variable being imputed itself. Primary exposure and outcome variables were included in the imputation model to preserve exposure–outcome associations but were not themselves imputed.

Inverse probability of treatment weighting (IPTW) was used as an additional robustness approach. Propensity scores were estimated using a generalized boosted model (GBM), and

stabilized weights were subsequently applied in weighted ordinal regression models. Covariate balance before and after weighting was evaluated using standardized mean differences (SMDs), with all covariates achieving an SMD below 0.10 after weighting, indicating adequate balance (Figure S5). To quantify the potential impact of unmeasured confounding, E-values were calculated according to the method proposed by VanderWeele and Ding. In addition, linear regression using heart rate as a negative control outcome was performed to assess potential residual unmeasured confounding. The distribution of stabilized IPTW weights was examined using the mean, standard deviation, median, range, and 1st and 99th percentiles. The number of participants whose weights were truncated was also recorded. Robust standard errors were used in IPTW models to account for weighting-induced variance estimation.

E-values were calculated to assess the minimum strength of association that an unmeasured confounder would need to have with both oral frailty and the health outcome, conditional on the measured covariates, to fully explain away the observed association. Because the health outcomes were common, odds ratios were not directly treated as risk ratios. Instead, OR estimates were approximately converted to the risk-ratio scale using the VanderWeele–Ding approximation for common outcomes, with $RR \approx \sqrt{OR}$. For estimates below the null, the reciprocal of the approximated RR was used so that the E-value was calculated in the direction away from the null. The E-value was calculated as E-value = $RR' + \sqrt{RR'（RR' - 1）}$, where RR' denotes the approximated risk ratio or its reciprocal, whichever is greater than 1. For confidence intervals, the bound closest to the null was used; when the confidence interval included the null, the corresponding confidence-limit E-value was set to 1.

Mediation analyses were performed within a causal mediation framework to investigate whether body composition and dietary/nutritional indicators mediated the association between oral frailty and physical health. Single-mediator analyses were conducted for body composition parameters, including skeletal muscle index, grip strength, and body fat percentage, as well as dietary and nutritional indicators, including E-DII-13, MAR, DBI-LBS, and CHEI-9. The quasi-Bayesian approach implemented in the *mediation* package, based on the framework developed by Imai et al., was used to estimate the average causal mediation effect, average direct effect, and mediation proportion, with 1,000 Monte Carlo simulations. Continuous mediators were standardized as z-scores prior to model fitting.

In addition, the prespecified chain mediation pathway from oral frailty to physical health through MAR and skeletal muscle index was examined using structural equation modeling in *lavaan*, with 5,000 bootstrap resamples used to estimate indirect effects. Detailed mediation estimates are presented in Tables S14–S16.

Predictive modeling was conducted using a nested logistic regression framework. Model A included baseline sociodemographic characteristics, namely age, sex, education, and income. Model B added OF-8-defined oral frailty to Model A. Model C further incorporated body composition parameters, including skeletal muscle index, grip strength, and body fat percentage. Model D additionally included dietary and nutritional indicators, including E-DII-13, MAR, DBI-LBS, and CHEI-9. Model discrimination was assessed using the area under the receiver operating characteristic curve (ROC-AUC), and optimism-corrected AUC estimates were obtained using 1,000 bootstrap resamples. Calibration was evaluated using calibration curves and the Hosmer–Lemeshow goodness-of-fit test. Clinical utility was examined using decision curve analysis. Differences in AUCs between nested models were compared using the DeLong test.

To further characterize the conditional dependency structure among oral frailty components, body composition, dietary indicators, and multidimensional health measures, an exploratory partial correlation network analysis was performed. Network nodes included all OF-8 items, skeletal muscle index, grip strength, E-DII-13, MAR, 24-hour dietary diversity, CHEI-9, and physical, mental, and social health scores. Continuous variables were standardized before analysis. A sparse Gaussian graphical model was estimated using the EBICglasso procedure implemented in the *bootnet* package. Network edges represent partial correlations between nodes after conditioning on all other variables in the network, and node centrality was quantified using one-step expected influence. This network analysis was intended as a hypothesis-generating supplementary analysis to visualize complex interrelationships among variables and was not interpreted as evidence of causal effects.

S2 Supplementary Table

Table S1 Baseline Characteristics According to OF-8-Defined Oral Frailty Status
See Table S1 in the supplementary materials.

Table S2 Baseline Characteristics According to SOFT-6-Defined Oral Frailty Status
See Table S2 in the supplementary materials.

Table S3 Brant Test for the Proportional Odds Assumption

Expose	Outcome	Brant *P*	Violate Hypothesis
OF-8	Overall health	0.057	No
OF-8	Physical health	<0.001	Yes
OF-8	Mental health	>0.999	No
OF-8	Social health	0.381	No
SOFT-6	Overall health	0.020	Yes
SOFT-6	Physical health	0.002	Yes
SOFT-6	Mental health	0.343	No
SOFT-6	Social health	0.399	No

Note: P<0.05, suggesting that the outcome violates the proportional superiority hypothesis; the corresponding results in the main text are supplemented with the Partial Proportional Superiority Model (PPO), maintaining the same key effect direction and significance.

Table S4 Full Nested Models for the Association Between OF-8-Defined Oral Frailty and Multidimensional Health

Outcome	Model	β (SE)	OR	95% CI	*P*
Overall health	M1	-0.581 (0.185)	0.559	0.389–0.804	0.002
	M2	-0.636 (0.194)	0.529	0.362–0.774	0.001
	M3	-0.590 (0.201)	0.554	0.374–0.822	0.003
	M4	-0.603 (0.202)	0.547	0.368–0.813	0.003
Physical health	M1	-0.720 (0.264)	0.487	0.290–0.817	0.006
	M2	-0.763 (0.280)	0.466	0.269–0.807	0.006
	M3	-0.716 (0.307)	0.489	0.268–0.891	0.020
	M4	-0.720 (0.308)	0.487	0.266–0.890	0.019
Mental health	M1	-0.336 (0.424)	0.715	0.311–1.639	0.428
	M2	-0.310 (0.432)	0.734	0.315–1.710	0.473
	M3	-0.260 (0.472)	0.771	0.306–1.946	0.582
	M4	-0.289 (0.477)	0.749	0.294–1.908	0.544
Social health	M1	-0.299 (0.181)	0.742	0.520–1.057	0.098
	M2	-0.397 (0.190)	0.672	0.463–0.975	0.036
	M3	-0.418 (0.200)	0.658	0.445–0.973	0.036

Outcome	Model	β (SE)	OR	95% CI	*P*
	M4	-0.426 (0.201)	0.653	0.441–0.968	0.034

Note: Model M1 adjusts for age and sex; Model M2 further adjusts for education and family annual income; Model M3 additionally adjusts for BMI, number of chronic diseases, smoking, alcohol consumption, and weekly physical activity; Model M4 further adjusts for dietary index.

Table S5 Multinomial Logistic Regression for Multidimensional Health Categories（OF-8）

Outcome	Model	Comparison	OR(95%CI)	*P*	*P*_Holm
Overall health	M1	health vs unhealth	0.347 (0.189–0.638)	<0.001	0.001
Overall health	M1	basic health vs unhealth	0.442 (0.248–0.789)	0.006	0.006
Overall health	M2	health vs unhealth	0.326 (0.172–0.617)	<0.001	0.001
Overall health	M2	basic health vs unhealth	0.474 (0.259–0.868)	0.016	0.016
Overall health	M3	health vs unhealth	0.334 (0.167–0.667)	0.002	0.004
Overall health	M3	basic health vs unhealth	0.487 (0.254–0.932)	0.030	0.030
Overall health	M4	health vs unhealth	0.326 (0.162–0.655)	0.002	0.003
Overall health	M4	basic health vs unhealth	0.484 (0.252–0.929)	0.029	0.029
Physical health	M1	health vs unhealth	0.379 (0.210–0.683)	0.001	0.003
Physical health	M1	basic health vs unhealth	0.162 (0.048–0.542)	0.003	0.003
Physical health	M2	health vs unhealth	0.379 (0.204–0.703)	0.002	0.004
Physical health	M2	basic health vs unhealth	0.171 (0.045–0.645)	0.009	0.009
Physical health	M3	health vs unhealth	0.389 (0.198–0.765)	0.006	0.006
Physical health	M3	basic health vs unhealth	0.080 (0.015–0.423)	0.003	0.006
Physical health	M4	health vs unhealth	0.389 (0.198–0.767)	0.006	0.010
Physical health	M4	basic health vs unhealth	0.082 (0.014–0.465)	0.005	0.010
Mental health	M1	health vs unhealth	0.323 (0.090–1.157)	0.083	0.085
Mental health	M1	basic health vs unhealth	0.163 (0.028–0.940)	0.042	0.085
Mental health	M2	health vs unhealth	0.339 (0.093–1.236)	0.101	0.106
Mental health	M2	basic health vs unhealth	0.175 (0.030–1.024)	0.053	0.106
Mental health	M3	health vs unhealth	0.372 (0.088–1.575)	0.179	0.179
Mental health	M3	basic health vs unhealth	0.149 (0.021–1.057)	0.057	0.114
Mental health	M4	health vs unhealth	0.326 (0.075–1.421)	0.136	0.136
Mental health	M4	basic health vs unhealth	0.126 (0.017–0.919)	0.041	0.082
Social health	M1	health vs unhealth	0.699 (0.430–1.139)	0.150	0.301
Social health	M1	basic health vs unhealth	1.030 (0.627–1.694)	0.907	0.907
Social health	M2	health vs unhealth	0.596 (0.356–0.999)	0.050	0.099
Social health	M2	basic health vs unhealth	0.943 (0.560–1.588)	0.826	0.826
Social health	M3	health vs unhealth	0.571 (0.328–0.995)	0.048	0.096
Social health	M3	basic health vs unhealth	0.902 (0.522–1.558)	0.711	0.711
Social health	M4	health vs unhealth	0.567 (0.323–0.993)	0.047	0.094

Social health	M4	basic health vs unhealth	0.925 (0.535–1.602)	0.782	0.782

Note：Using "unhealthy" as the reference category, Model M1 adjusted for age and sex; Model M2 further adjusted for education and income; Model M3 additionally adjusted for BMI, number of chronic diseases, smoking, alcohol consumption, and physical activity; and Model M4 further adjusted for dietary inflammation index and 24-hour dietary diversity based on Model M3. An OR> 1 indicates a higher likelihood that individuals with oral frailty are in a corresponding higher health status, while an OR <1 indicates a lower likelihood.

Table S6 Full Nested Models for the Association Between SOFT-6-Defined Oral Frailty and Multidimensional Health

Outcome	Exposure	Model	OR	95%CI_low	95%CI_high	*P*
Overall health	SOFT-6_yes	M1	0.509	0.356	0.726	<0.001
Overall health	SOFT-6_yes	M2	0.534	0.369	0.774	0.001
Overall health	SOFT-6_yes	M3	0.564	0.384	0.828	0.003
Overall health	SOFT-6_yes	M4	0.564	0.383	0.830	0.004
Physical health	SOFT-6_yes	M1	0.388	0.234	0.644	<0.001
Physical health	SOFT-6_yes	M2	0.398	0.234	0.677	0.001
Physical health	SOFT-6_yes	M3	0.441	0.250	0.779	0.005
Physical health	SOFT-6_yes	M4	0.444	0.251	0.786	0.005
Mental health	SOFT-6_yes	M1	0.177	0.060	0.523	0.002
Mental health	SOFT-6_yes	M2	0.197	0.066	0.586	0.003
Mental health	SOFT-6_yes	M3	0.197	0.061	0.635	0.007
Mental health	SOFT-6_yes	M4	0.204	0.063	0.666	0.008
Social health	SOFT-6_yes	M1	0.784	0.555	1.106	0.165
Social health	SOFT-6_yes	M2	0.807	0.563	1.155	0.241
Social health	SOFT-6_yes	M3	0.841	0.579	1.223	0.365
Social health	SOFT-6_yes	M4	0.861	0.590	1.255	0.436

Note: Model M1 adjusts for age and sex; Model M2 further adjusts for education and family annual income; Model M3 additionally adjusts for BMI, number of chronic diseases, smoking, alcohol consumption, and weekly physical activity; Model M4 further adjusts for dietary index.

Table S7 Multinomial Logistic Regression for Multidimensional Health Categories（SOFT-6）

Outcome	Model	Comparison	OR_95%CI	*P*	*P*_Holm
Overall health	M1	health vs unhealth	0.317 (0.177–0.568)	<0.001	<0.001
Overall health	M1	basic health vs unhealth	0.397 (0.229–0.688)	<0.001	<0.001
Overall health	M2	health vs unhealth	0.321 (0.174–0.593)	<0.001	<0.001
Overall health	M2	basic health vs unhealth	0.378 (0.212–0.673)	<0.001	<0.001
Overall health	M3	health vs unhealth	0.346 (0.180–0.664)	0.001	0.003
Overall health	M3	basic health vs unhealth	0.377 (0.204–0.697)	0.002	0.003

Outcome	Model	Comparison	OR_95%CI	***P***	***P*_Holm**
Overall health	M4	health vs unhealth	0.348 (0.180–0.672)	0.002	0.003
Overall health	M4	basic health vs unhealth	0.379 (0.204–0.703)	0.002	0.003
Physical health	M1	health vs unhealth	0.378 (0.220–0.649)	<0.001	<0.001
Physical health	M1	basic health vs unhealth	0.928 (0.263–3.265)	0.907	0.907
Physical health	M2	health vs unhealth	0.382 (0.216–0.674)	<0.001	0.002
Physical health	M2	basic health vs unhealth	0.788 (0.202–3.077)	0.732	0.732
Physical health	M3	health vs unhealth	0.411 (0.223–0.758)	0.004	0.009
Physical health	M3	basic health vs unhealth	0.579 (0.126–2.669)	0.484	0.484
Physical health	M4	health vs unhealth	0.416 (0.225–0.771)	0.005	0.011
Physical health	M4	basic health vs unhealth	0.536 (0.109–2.648)	0.444	0.444
Mental health	M1	health vs unhealth	0.142 (0.032–0.633)	0.010	0.021
Mental health	M1	basic health vs unhealth	0.592 (0.070–5.031)	0.631	0.631
Mental health	M2	health vs unhealth	0.162 (0.036–0.732)	0.018	0.036
Mental health	M2	basic health vs unhealth	0.634 (0.074–5.449)	0.678	0.678
Mental health	M3	health vs unhealth	0.155 (0.029–0.842)	0.031	0.062
Mental health	M3	basic health vs unhealth	0.508 (0.050–5.160)	0.567	0.567
Mental health	M4	health vs unhealth	0.144 (0.026–0.807)	0.028	0.055
Mental health	M4	basic health vs unhealth	0.459 (0.044–4.771)	0.514	0.514
Social health	M1	health vs unhealth	0.750 (0.466–1.208)	0.237	0.474
Social health	M1	basic health vs unhealth	1.062 (0.656–1.717)	0.808	0.808
Social health	M2	health vs unhealth	0.757 (0.460–1.247)	0.274	0.549
Social health	M2	basic health vs unhealth	0.995 (0.603–1.640)	0.983	0.983
Social health	M3	health vs unhealth	0.772 (0.453–1.318)	0.343	0.687
Social health	M3	basic health vs unhealth	0.952 (0.563–1.610)	0.854	0.854
Social health	M4	health vs unhealth	0.806 (0.468–1.387)	0.436	0.872
Social health	M4	basic health vs unhealth	1.002 (0.588–1.706)	0.995	0.995

Note：Using "unhealthy" as the reference category, Model M1 adjusted for age and sex; Model M2 further adjusted for education and income; Model M3 additionally adjusted for BMI, number of chronic diseases, smoking, alcohol consumption, and physical activity; and Model M4 further adjusted for dietary inflammation index and 24-hour dietary diversity based on Model M3. An OR> 1 indicates a higher likelihood that individuals with oral frailty are in a corresponding higher health status, while an OR <1 indicates a lower likelihood.

Table S8 IPTW-Weighted Associations Between OF-8-Defined Oral Frailty and Multidimensional Health

Outcome	Weighted OR	95% CI	***P***
Overall health	0.554	0.382-0.802	0.002

Physical health	0.571	0.345-0.944	0.029
Mental health	0.862	0.381-1.950	0.721
Social health	0.672	0.468-0.965	0.031

Note: The propensity scores were estimated using the Generalized Boosting Model (GBM); stable weights were employed; after covariate weighting, all standardized mean differences (SMDs) were <0.10 (see Figure S5).

Table S9 E-Value Analysis for Unmeasured Confounding

Outcome	OR (95% CI)	Similar RR	E-value (point)	E-value (95% CI _high)
Overall health	0.557 (0.376—0.826)	0.747	2.01	1.43
Physical health	0.494 (0.271—0.900)	0.703	2.20	1.29
Mental health	0.814 (0.321—2.065)	0.902	1.45	1.00
Social health	0.666 (0.451—0.984)	0.816	1.75	1.10

Note: The E-value was calculated based on the conversion of OR to approximate RR by VanderWeele and Ding (2017). A higher E-value indicates that stronger unmeasured confounding is required to explain the observed association. The E-value for the upper limit of the mental health CI is 1.00, suggesting that this association is highly sensitive to unmeasured confounding.

Table S10 Negative Control Outcome Analysis Using Resting Heart Rate

Model	β	SE	t	*P*
HR ~ OF-8 + M3	0.220	0.136	1.616	0.107

Note: HR should not be directly affected by OF-8; as a placebo-like outcome test, no unmeasured confounding was detected. The null hypothesis was not rejected (P=0.107), supporting that the primary analysis does not reveal significant unmeasured confounding.

Table S11 Sensitivity Analysis Excluding Participants with Extreme Energy Intake

Exposure	Outcome	OR	95% CI	*P*
OF-8	Overall health	0.629	0.412—0.962	0.033
OF-8	Physical health	0.579	0.305—1.099	0.095
OF-8	Mental health	0.754	0.254—2.241	0.611
OF-8	Social health	0.669	0.440—1.017	0.060

SOFT-6	Overall health	0.658	0.435—0.997	0.049
SOFT-6	Physical health	0.466	0.252—0.864	0.015
SOFT-6	Mental health	0.053	0.006—0.446	0.007
SOFT-6	Social health	1.003	0.668—1.505	0.989

Note: After excluding individuals with a daily total energy intake below 800 kcal or above 4200 kcal, the direction of association remained consistent with the principal analysis.

Table S12 Multiple linear regression analysis of the composite score for oral frailty and health

Model	Exposure	Outcome	beta	95%CI_low	95%CI_high	*P*
M1	OF8_yes	Overall health score	-1.898	-3.651	-0.144	0.034
M2	OF8_yes	Overall health score	-2.302	-4.124	-0.481	0.013
M3	OF8_yes	Overall health score	-1.520	-3.264	0.223	0.087
M4	OF8_yes	Overall health score	-1.529	-3.262	0.205	0.084
M1	OF8_yes	Physical health score	-1.306	-2.235	-0.377	0.006
M2	OF8_yes	Physical health score	-1.472	-2.431	-0.513	0.003
M3	OF8_yes	Physical health score	-0.935	-1.861	-0.009	0.048
M4	OF8_yes	Physical health score	-0.948	-1.872	-0.025	0.044
M1	OF8_yes	Mental health score	-0.349	-0.904	0.206	0.217
M2	OF8_yes	Mental health score	-0.416	-0.995	0.163	0.158
M3	OF8_yes	Mental health score	-0.306	-0.880	0.268	0.295
M4	OF8_yes	Mental health score	-0.300	-0.872	0.272	0.304
M1	OF8_yes	Social health score	-0.243	-1.100	0.614	0.578
M2	OF8_yes	Social health score	-0.414	-1.289	0.461	0.353
M3	OF8_yes	Social health score	-0.279	-1.149	0.590	0.528
M4	OF8_yes	Social health score	-0.281	-1.150	0.588	0.525
M1	SOFT-6_yes	Overall health score	-3.812	-5.499	-2.126	<0.001
M2	SOFT-6_yes	Overall health score	-3.664	-5.422	-1.906	<0.001
M3	SOFT-6_yes	Overall health score	-2.902	-4.582	-1.222	0.001
M4	SOFT-6_yes	Overall health score	-2.759	-4.443	-1.075	0.001
M1	SOFT-6_yes	Physical health score	-2.173	-3.067	-1.280	<0.001
M2	SOFT-6_yes	Physical health score	-2.146	-3.071	-1.222	<0.001
M3	SOFT-6_yes	Physical health score	-1.672	-2.563	-0.780	<0.001
M4	SOFT-6_yes	Physical health score	-1.639	-2.535	-0.743	<0.001
M1	SOFT-6_yes	Mental health score	-1.054	-1.589	-0.520	<0.001
M2	SOFT-6_yes	Mental health score	-0.976	-1.535	-0.417	0.001
M3	SOFT-6_yes	Mental health score	-0.835	-1.388	-0.281	0.003
M4	SOFT-6_yes	Mental health score	-0.780	-1.336	-0.223	0.006
M1	SOFT-6_yes	Social health score	-0.585	-1.421	0.252	0.170

Model	Exposure	Outcome	beta	95%CI_low	95%CI_high	*P*
M2	SOFT-6_yes	Social health score	-0.542	-1.396	0.312	0.213
M3	SOFT-6_yes	Social health score	-0.396	-1.241	0.450	0.358
M4	SOFT-6_yes	Social health score	-0.341	-1.192	0.511	0.432

Table S13 Results of the dose-response relationship test between oral frailty scores and health scores

Exposure	Outcome	Overall_*P*	Nonlinear_*P*
OF-8	Overall health score	0.002	0.603
OF-8	Physical health score	<0.001	0.381
OF-8	Mental health score	0.003	0.226
OF-8	Social health score	0.588	0.591
SOFT-6	Overall health score	<0.001	0.652
SOFT-6	Physical health score	<0.001	0.275
SOFT-6	Mental health score	0.006	0.865
SOFT-6	Social health score	0.607	0.520

Table S14 Exploratory Mediation Analyses Through Body Composition Indicators

Mediation	ACME (95% CI)	ADE (95% CI)	Ratio	*P* (ACME)
SMI	-0.028 (-0.120, 0.036)	-0.927 (-1.756, -0.092)	2.9% (-6.0, 21.4)	0.494
The power of gripping	-0.165 (-0.376, 0.031)	-0.724 (-1.632, 0.104)	18.5% (-8.5, 117.6)	0.110
Body fat percentage	-0.027 (-0.117, 0.038)	-0.928 (-1.765, -0.067)	2.8% (-6.2, 19.2)	0.486

Note: ACME = Average Causal Mediation Effect; ADE = Average Direct Effect. Based on 1,000 Monte Carlo simulations, all P-values of ACME were>0.05, indicating that body composition does not constitute a mediating pathway in the association between OF-8 and physical health.

Table S15 Exploratory Mediation Analyses Through Dietary Indicators

Mediation	ACME (95% CI)	ADE (95% CI)	Ratio	*P* (ACME)
E-DII13	0.011 (-0.048, 0.092)	-0.858 (-1.722, -0.007)	-1.3% (-17.6, 14.6)	0.722
MAR	0.005 (-0.066, 0.089)	-0.852 (-1.642, -0.039)	-0.6% (-19.6, 17.1)	0.898
DBI-LBS	-0.002 (-0.086, 0.075)	-0.858 (-1.726, -0.025)	0.2% (-17.2,	0.984

			15.1)	
CHEI-9	0.074 (-0.019, 0.223)	-0.968 (-1.905, -0.122)	-8.3% (-64.7, 6.6)	0.146

Note: None of the four dietary indices demonstrated significant mediating effects; similar to body composition findings, this suggests that diet is not the primary mediator between OF-8 and physical health in the study population.

Table S16 Exploratory Risk Stratification Model Performance

pathway	Effect	95% CI	Significant
Total effect	-0.854	-1.666 ~ 0.039	No
Direct effect	-0.828	-1.641 ~ 0.055	No
Only the indirect effect of MAR	0.004	-0.068 ~ 0.088	No
Only the SMI indirect effect	-0.030	-0.117 ~ 0.033	No
Chain-type (MAR→SMI) indirect	0.0002	-0.003 ~ 0.005	No

Note: Lavaan performed 5,000 bootstrap iterations. The point estimate for the chain-mediated effect is close to 0, indicating that the mechanism of "nutritional deficiency mediating sarcopenia" is unlikely to hold true in this cross-sectional sample.

Table S17 Performance of the nested Logistic prediction model and the DeLong test

Model	Original AUC	Adjust AUC	Calibration slope	Brier	DeLong vs Previous model *P*
A (demography)	0.732	0.705	0.759	0.142	—
B = A + OF-8	0.764	0.734	0.767	0.139	0.095
C = B + Body Composition	0.778	0.746	0.757	0.135	0.292
D = C + Food	0.780	0.741	0.730	0.137	0.624

Note: 1,000 bootstrap iterations correct for optimism bias. "DeLong P" represents the AUC difference test between this model and the previous model.

Table S18 Fitting metrics for Potential Profile Analysis (LPA) (based on the comprehensive health score and three-dimensional subscores)

Categories	LogLik	AIC	BIC	SABIC	Entropy	BLRT *P*
2	-1770.4	3560.7	3601.9	3570.1	0.974	0.010
3	-1698.8	3425.6	3483.2	3438.8	0.958	0.010
4	-1698.4	3432.8	3506.9	3449.8	0.577	0.634

Note: The BIC value is lowest for Category 3 with an Entropy>0.9; the BLRT test shows no significance for Category 4, hence the three potential profiles are ultimately selected.

Table S19 Health–Body–Diet Network Centrality (Impact 1-step)

Node	1-step Influence
Dietary Diversity (24 hours)	0.986
Grip strength (kg)	0.642
Physical health score	0.622
Social health score	0.440
CHEI-9 score	0.432
Mental health score	0.429
SMI	0.372
MAR	-0.143
E-DII13	-0.643

Note: A 1-step influence assessment based on Bayesian networks. Dietary diversity, grip strength, and physical health scores ranked top three, consistent with the mediating structure where the "diet-muscle-skeleton" pathway was not significantly independent but interrelated.

Table S20 Missing Data Pattern and Multiple Imputation Details

Variable	Missing percentage (%)
Probable_sarc	12.1
Handgrip_max	11.2
Tooth_number	11
Diet_div_24h	7.3
NRF_simple	6.2
CHEI_total	5.9

Variable	Missing percentage (%)
Family_income	5.3
Energy_adequacy	4.8
Diet_div_ffq	3.5
EDII13	2.9
MAR	2.9
Prot_density	2.9
NaK_ratio	2.9
AOXI	2.9
BMI_body	2

Table S21 Complete-case analyses compared with multiple-imputation analyses

Exposure definition	Outcome	Model	Multiple-imputation analysis OR (95% CI), P	Complete-case analysis OR (95% CI), P	Complete-case N
OF-8	Overall health	M1	0.559 (0.389–0.804), P=0.002	0.559 (0.389–0.804), P=0.002	454
OF-8	Overall health	M2	0.529 (0.362–0.774), P=0.001	0.529 (0.362–0.774), P=0.001	429
OF-8	Overall health	M3	0.557 (0.375–0.825), P=0.004	0.537 (0.360–0.799), P=0.002	420
OF-8	Overall health	M4	0.551 (0.370–0.818), P=0.003	0.600 (0.399–0.904), P=0.015	389
OF-8	Physical health	M1	0.487 (0.290–0.817), P=0.006	0.487 (0.290–0.817), P=0.006	454
OF-8	Physical health	M2	0.466 (0.269–0.807), P=0.006	0.466 (0.269–0.807), P=0.006	429
OF-8	Physical health	M3	0.489 (0.268–0.892), P=0.020	0.472 (0.257–0.867), P=0.015	420
OF-8	Physical health	M4	0.482 (0.263–0.882), P=0.018	0.549 (0.291–1.033), P=0.063	389
OF-8	Mental health	M1	0.715 (0.311–1.639), P=0.428	0.715 (0.311–1.639), P=0.428	454
OF-8	Mental health	M2	0.734 (0.315–1.710), P=0.473	0.734 (0.315–1.710), P=0.473	429
OF-8	Mental health	M3	0.786 (0.311–1.988), P=0.611	0.739 (0.276–1.976), P=0.546	420
OF-8	Mental health	M4	0.742 (0.290–1.900), P=0.534	0.770 (0.263–2.253), P=0.634	389
OF-8	Social health	M1	0.742 (0.520–1.057), P=0.098	0.742 (0.520–1.057), P=0.098	454
OF-8	Social health	M2	0.672 (0.463–0.975), P=0.036	0.672 (0.463–0.975), P=0.036	429
OF-8	Social health	M3	0.664 (0.449–0.981), P=0.040	0.635 (0.428–0.942), P=0.024	420
OF-8	Social health	M4	0.662 (0.447–0.980), P=0.039	0.666 (0.443–1.001), P=0.051	389
SOFT-6	Overall health	M1	0.509 (0.356–0.726), P=<0.001	0.509 (0.356–0.726), P=<0.001	454
SOFT-6	Overall health	M2	0.534 (0.369–0.774), P=<0.001	0.534 (0.369–0.774), P=<0.001	429
SOFT-6	Overall health	M3	0.565 (0.385–0.830), P=0.004	0.559 (0.379–0.824), P=0.003	420
SOFT-6	Overall health	M4	0.567 (0.385–0.836), P=0.004	0.631 (0.422–0.943), P=0.025	389
SOFT-6	Physical health	M1	0.388 (0.234–0.644), P=<0.001	0.388 (0.234–0.644), P=<0.001	454
SOFT-6	Physical health	M2	0.398 (0.234–0.677), P=<0.001	0.398 (0.234–0.677), P=<0.001	429
SOFT-6	Physical health	M3	0.441 (0.250–0.779), P=0.005	0.445 (0.251–0.789), P=0.006	420
SOFT-6	Physical health	M4	0.448 (0.253–0.793), P=0.006	0.477 (0.260–0.875), P=0.017	389
SOFT-6	Mental health	M1	0.177 (0.060–0.523), P=0.002	0.177 (0.060–0.523), P=0.002	454

SOFT-6	Mental health	M2	0.197 (0.066–0.586), P=0.003	0.197 (0.066–0.586), P=0.003	429
SOFT-6	Mental health	M3	0.198 (0.061–0.642), P=0.007	0.227 (0.068–0.755), P=0.016	420
SOFT-6	Mental health	M4	0.205 (0.063–0.671), P=0.009	0.106 (0.021–0.539), P=0.007	389
SOFT-6	Social health	M1	0.784 (0.555–1.106), P=0.165	0.784 (0.555–1.106), P=0.165	454
SOFT-6	Social health	M2	0.807 (0.563–1.155), P=0.241	0.807 (0.563–1.155), P=0.241	429
SOFT-6	Social health	M3	0.844 (0.581–1.227), P=0.375	0.852 (0.584–1.242), P=0.404	420
SOFT-6	Social health	M4	0.864 (0.592–1.261), P=0.448	0.957 (0.645–1.420), P=0.827	389

Table S22 Threshold-specific partial proportional odds models for outcomes violating the proportional odds assumption

Exposure	Outcome	Model	threshold	OR_95CI	*P*
SOFT-6	Physical health	M1	≥Basic healthy vs Unhealthy	0.522 (0.320–0.852)	0.009
SOFT-6	Physical health	M1	Healthy vs ≤Basic healthy	0.415 (0.256–0.672)	<0.001
SOFT-6	Physical health	M2	≥Basic healthy vs Unhealthy	0.550 (0.336–0.901)	0.018
SOFT-6	Physical health	M2	Healthy vs ≤Basic healthy	0.437 (0.268–0.710)	<0.001
SOFT-6	Physical health	M3	≥Basic healthy vs Unhealthy	0.620 (0.373–1.030)	0.065
SOFT-6	Physical health	M3	Healthy vs ≤Basic healthy	0.492 (0.298–0.813)	0.006
SOFT-6	Physical health	M4	≥Basic healthy vs Unhealthy	0.625 (0.375–1.043)	0.072
SOFT-6	Physical health	M4	Healthy vs ≤Basic healthy	0.496 (0.299–0.823)	0.007
SOFT-6	Overall health	M1	≥Basic healthy vs Unhealthy	1.723 (1.154–2.573)	0.008
SOFT-6	Overall health	M1	Healthy vs ≤Basic healthy	0.261 (0.175–0.390)	<0.001
SOFT-6	Overall health	M2	≥Basic healthy vs Unhealthy	1.838 (1.227–2.753)	0.003
SOFT-6	Overall health	M2	Healthy vs ≤Basic healthy	0.279 (0.186–0.416)	<0.001
SOFT-6	Overall health	M3	≥Basic healthy vs Unhealthy	1.983 (1.298–3.031)	0.002
SOFT-6	Overall health	M3	Healthy vs ≤Basic healthy	0.301 (0.198–0.457)	<0.001
SOFT-6	Overall health	M4	≥Basic healthy vs Unhealthy	1.981 (1.294–3.033)	0.002
SOFT-6	Overall health	M4	Healthy vs ≤Basic healthy	0.300 (0.197–0.458)	<0.001
OF-8	Physical health	M1	≥Basic healthy vs Unhealthy	0.597 (0.358–0.994)	0.047
OF-8	Physical health	M1	Healthy vs ≤Basic healthy	0.531 (0.320–0.881)	0.014
OF-8	Physical health	M2	≥Basic healthy vs Unhealthy	0.575 (0.346–0.956)	0.033
OF-8	Physical health	M2	Healthy vs ≤Basic healthy	0.512 (0.309–0.848)	0.009
OF-8	Physical health	M3	≥Basic healthy vs Unhealthy	0.669 (0.396–1.127)	0.131
OF-8	Physical health	M3	Healthy vs ≤Basic healthy	0.595 (0.354–1.000)	0.050
OF-8	Physical health	M4	≥Basic healthy vs Unhealthy	0.671 (0.398–1.132)	0.135
OF-8	Physical health	M4	Healthy vs ≤Basic healthy	0.597 (0.355–1.004)	0.052

Table S23 Distribution of multidimensional health categories by oral frailty status

Exposure	Outcome	Exposure level	Health level	n	Percent
OF-8	Overall health	no	Unhealthy	20	10.8
OF-8	Overall health	no	Basic healthy	93	50
OF-8	Overall health	no	Healthy	73	39.2
OF-8	Overall health	yes	Unhealthy	70	26.1
OF-8	Overall health	yes	Basic healthy	124	46.3
OF-8	Overall health	yes	Healthy	74	27.6
OF-8	Physical health	no	Unhealthy	17	9.1
OF-8	Physical health	no	Basic healthy	8	4.3
OF-8	Physical health	no	Healthy	161	86.6
OF-8	Physical health	yes	Unhealthy	66	24.6
OF-8	Physical health	yes	Basic healthy	7	2.6
OF-8	Physical health	yes	Healthy	195	72.8
OF-8	Mental health	no	Unhealthy	3	1.6
OF-8	Mental health	no	Basic healthy	6	3.2
OF-8	Mental health	no	Healthy	177	95.2
OF-8	Mental health	yes	Unhealthy	14	5.2
OF-8	Mental health	yes	Basic healthy	5	1.9
OF-8	Mental health	yes	Healthy	249	92.9
OF-8	Social health	no	Unhealthy	44	23.7
OF-8	Social health	no	Basic healthy	61	32.8
OF-8	Social health	no	Healthy	81	43.5
OF-8	Social health	yes	Unhealthy	75	28
OF-8	Social health	yes	Basic healthy	103	38.4
OF-8	Social health	yes	Healthy	90	33.6
SOFT-6	Overall health	no	Unhealthy	24	11.1
SOFT-6	Overall health	no	Basic healthy	109	50.5
SOFT-6	Overall health	no	Healthy	83	38.4
SOFT-6	Overall health	yes	Unhealthy	66	27.7
SOFT-6	Overall health	yes	Basic healthy	108	45.4
SOFT-6	Overall health	yes	Healthy	64	26.9
SOFT-6	Physical health	no	Unhealthy	23	10.6
SOFT-6	Physical health	no	Basic healthy	4	1.9
SOFT-6	Physical health	no	Healthy	189	87.5
SOFT-6	Physical health	yes	Unhealthy	60	25.2
SOFT-6	Physical health	yes	Basic healthy	11	4.6
SOFT-6	Physical health	yes	Healthy	167	70.2
SOFT-6	Mental health	no	Unhealthy	2	0.9
SOFT-6	Mental health	no	Basic healthy	2	0.9
SOFT-6	Mental health	no	Healthy	212	98.1
SOFT-6	Mental health	yes	Unhealthy	15	6.3
SOFT-6	Mental health	yes	Basic healthy	9	3.8
SOFT-6	Mental health	yes	Healthy	214	89.9
SOFT-6	Social health	no	Unhealthy	53	24.5
SOFT-6	Social health	no	Basic healthy	72	33.3
SOFT-6	Social health	no	Healthy	91	42.1
SOFT-6	Social health	yes	Unhealthy	66	27.7
SOFT-6	Social health	yes	Basic healthy	92	38.7
SOFT-6	Social health	yes	Healthy	80	33.6

S3 Supplementary Figures

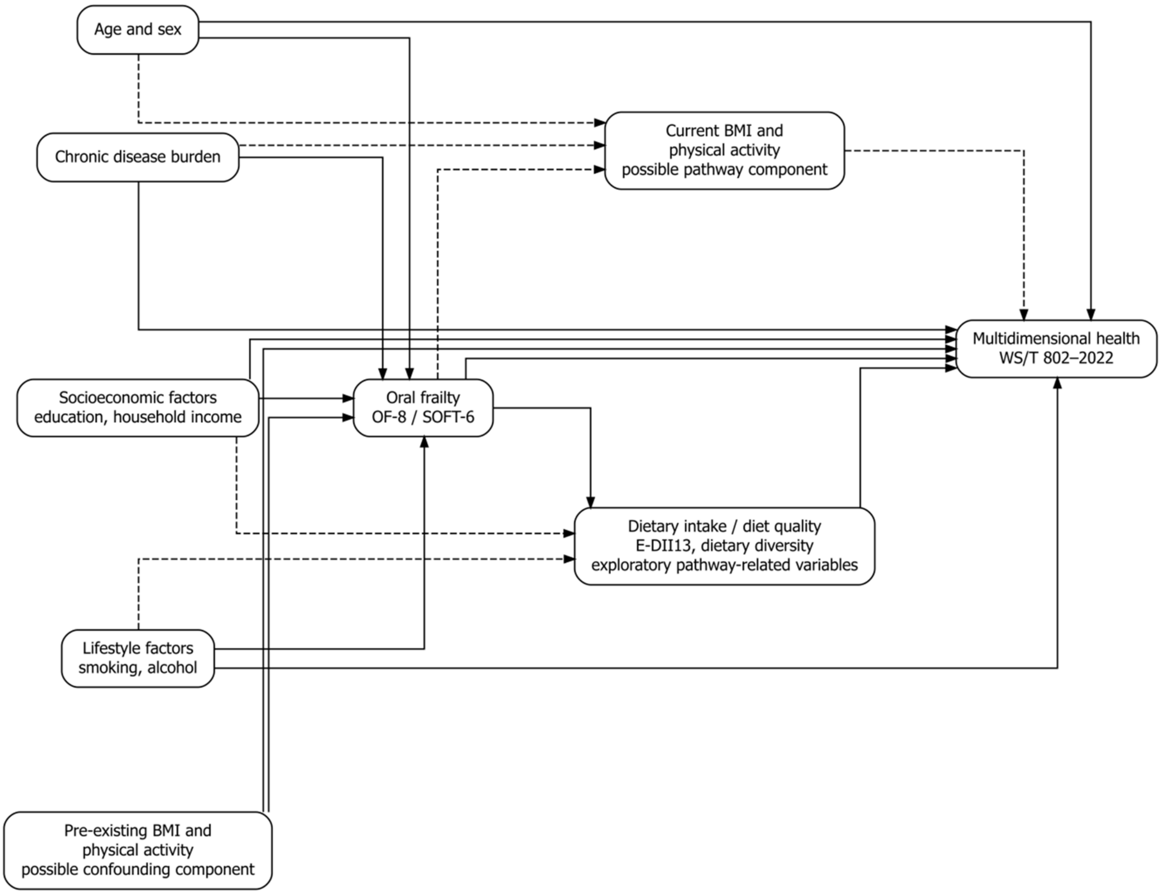

Figure S1 Directed acyclic graph for covariate selection.
Note:Age, sex, socioeconomic factors, lifestyle factors, chronic disease burden, and pre-existing BMI and physical activity were considered potential confounding structures. Dietary intake and diet quality were considered exploratory pathway-related variables. BMI and physical activity were regarded as mixed-role variables because cross-sectional measurements may reflect both pre-existing confounding components and pathway-related components. Dashed arrows indicate uncertain or pathway-related mechanisms.

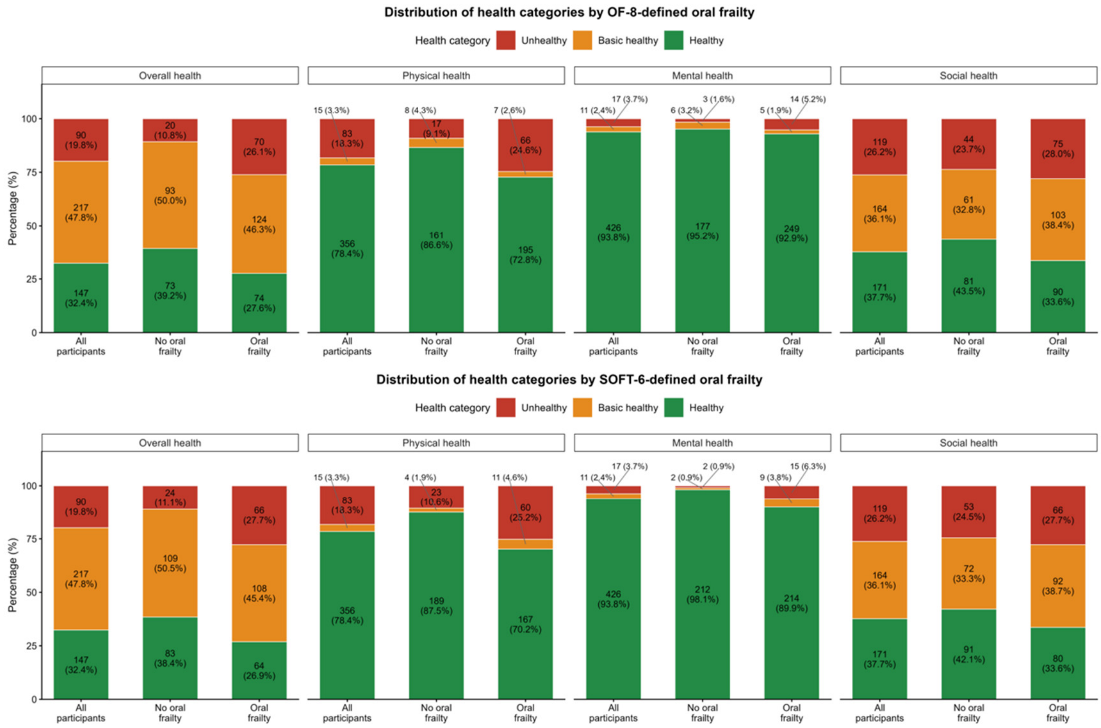

Figure S2 Distribution of health categories according to oral frailty status defined by OF-8 and SOFT-6

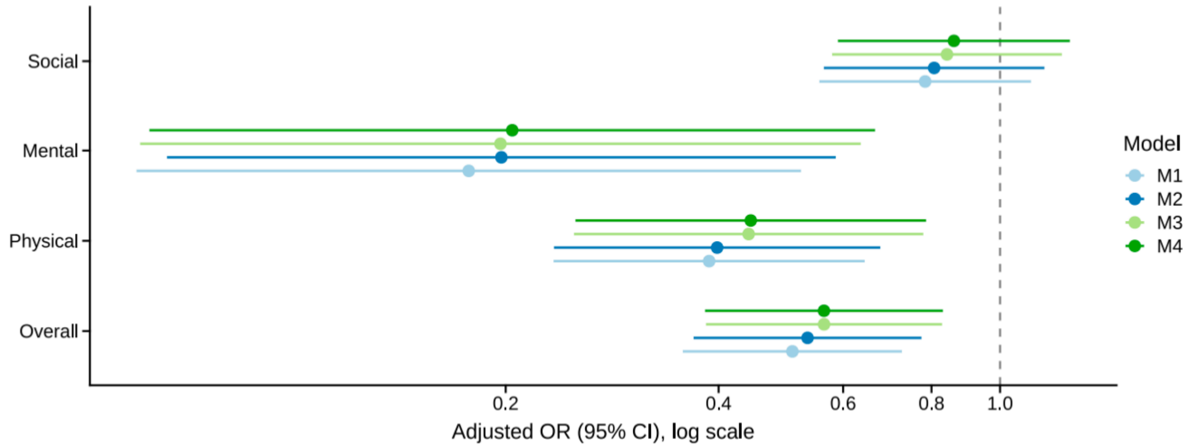

Figure S3 Adjusted associations between SOFT-6-defined oral frailty and multidimensional health

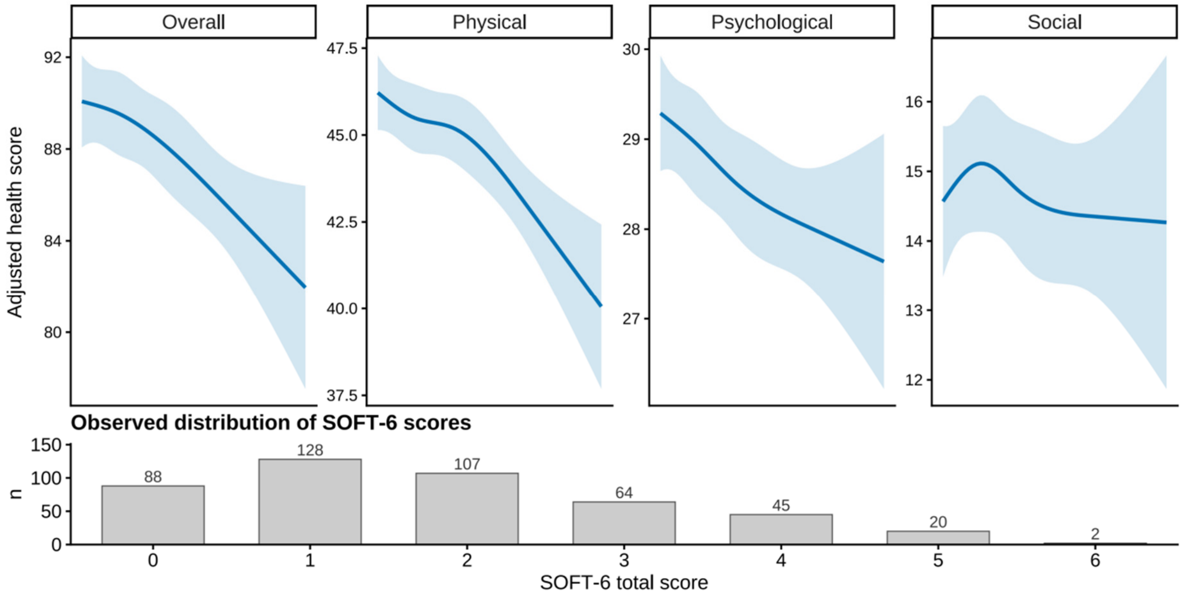

FigureS4 Dose-Response Associations Between SOFT-6 Score and Health Score

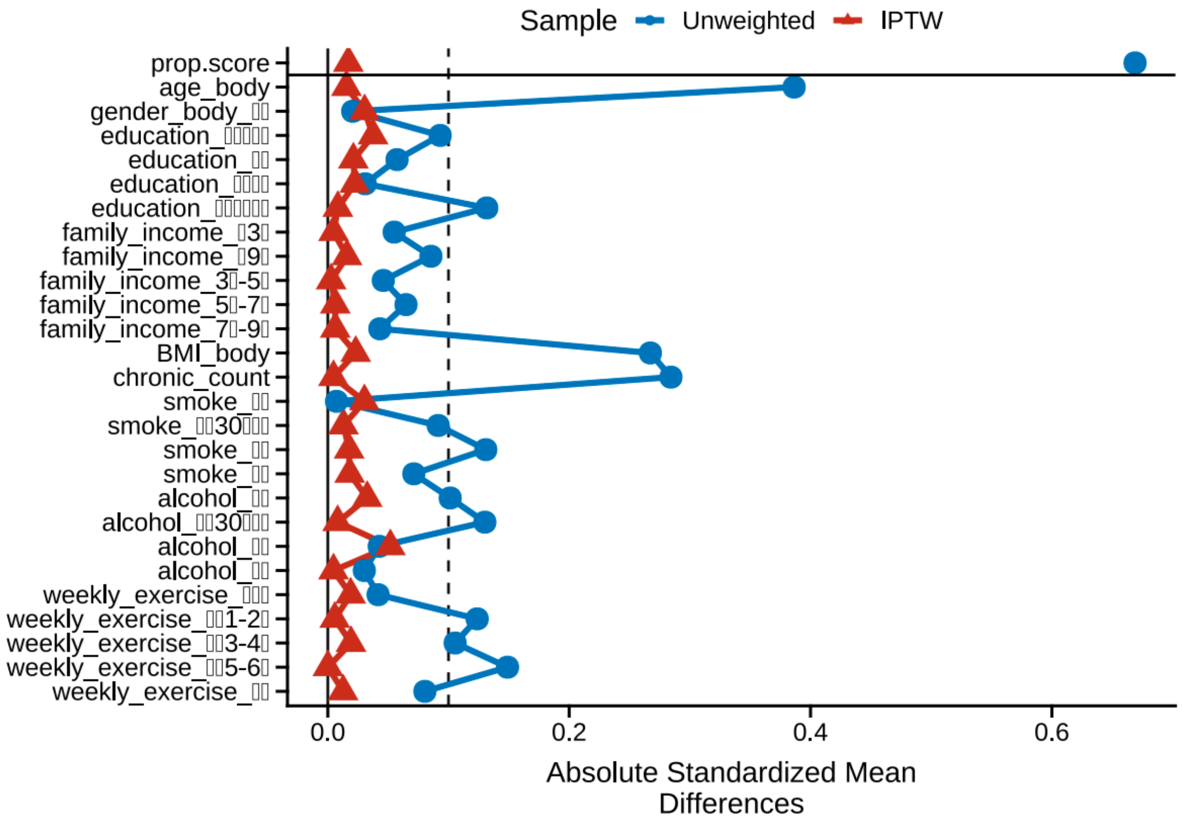

Figure S5 Equilibrium of covariates before and after IPTW weighting (Love plot)

Note:Absolute standardized mean difference (|SMD|). For pre-weighted partial covariates, |SMD|> 0.10; for all post-weighted covariates, |SMD| <0.10, indicating good balance.

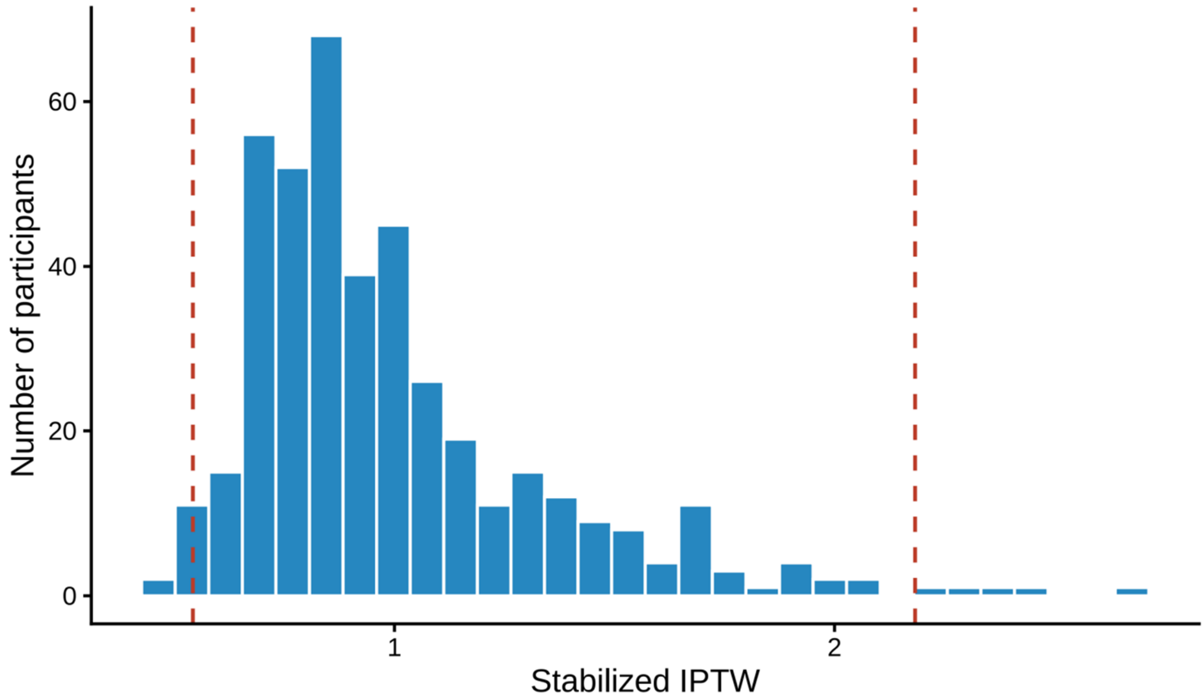

Figure S6 Distribution of stabilized of IPTW

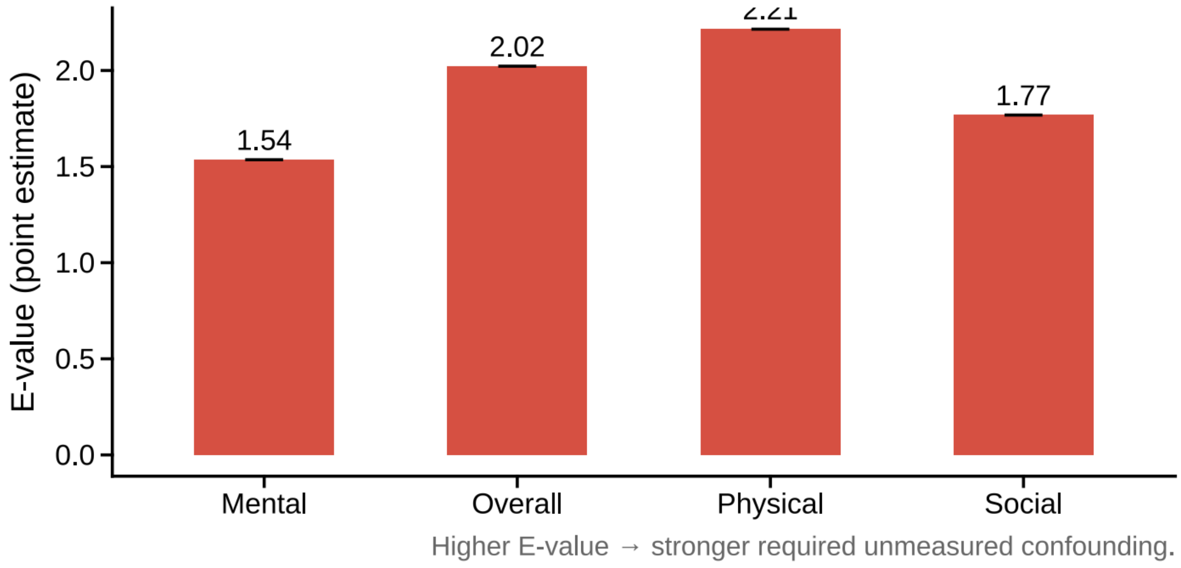

Figure S7 E-value scatter plot (based on M3-adjusted OR)

Note:Point estimates of E-values and the upper limits of 95% confidence intervals (CI) corresponding to the four-dimensional health outcomes. A combined health and physical health E-value ≥1.43 (CI) indicates that the primary conclusion remains relatively robust despite unmeasured confounders.

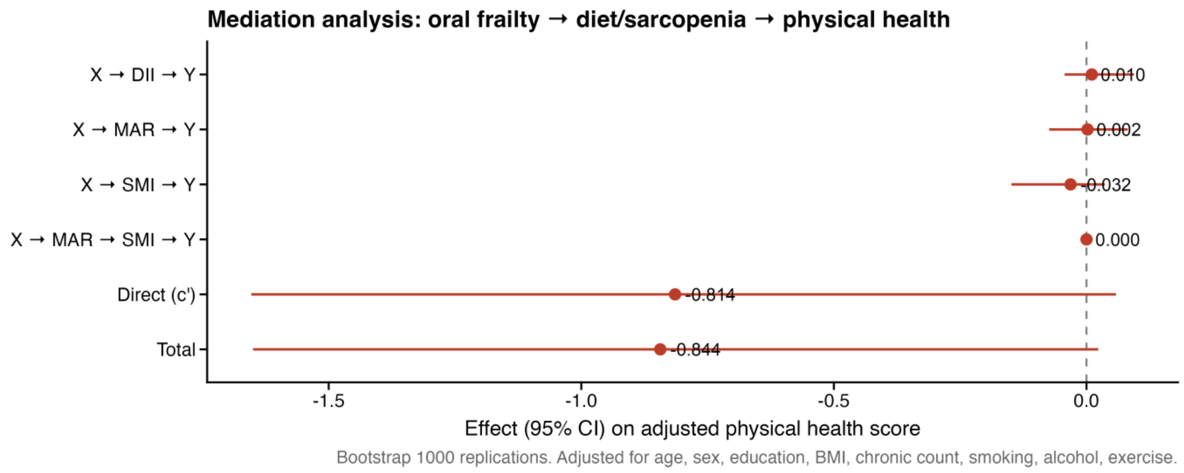

Figure S8 Forest plot of the mediating pathway from oral frailty to systemic health (based on 5,000 bootstrap iterations with adjustment for M3 covariates)

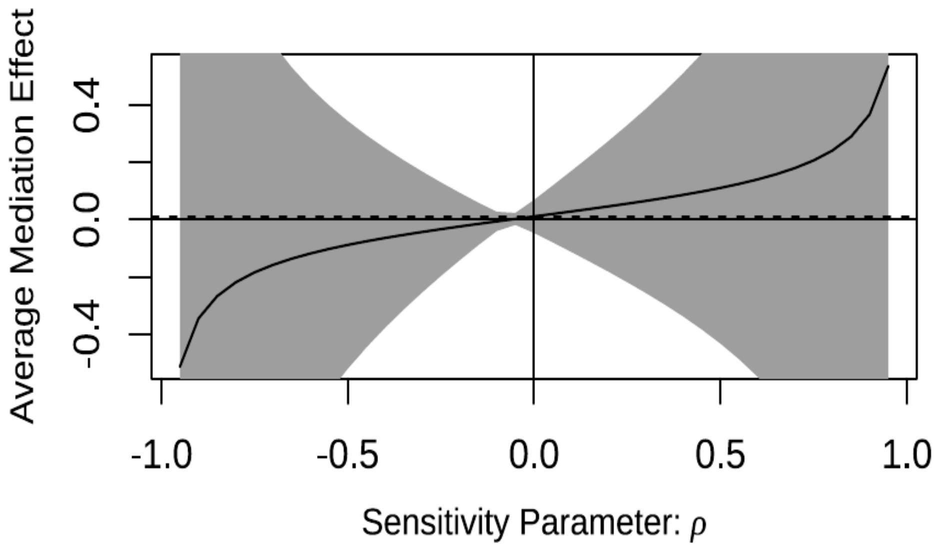

Figure S9 Sensitivity analysis of the mediative pathway in the dietary pro-inflammatory index E-DII13

Note：it shows the robustness of the E-DII13 mediating pathway under different untested confounding assumptions; ACME crosses zero when ρ approaches 0, indicating that the mediating effect is highly unstable in the presence of untested confounding.

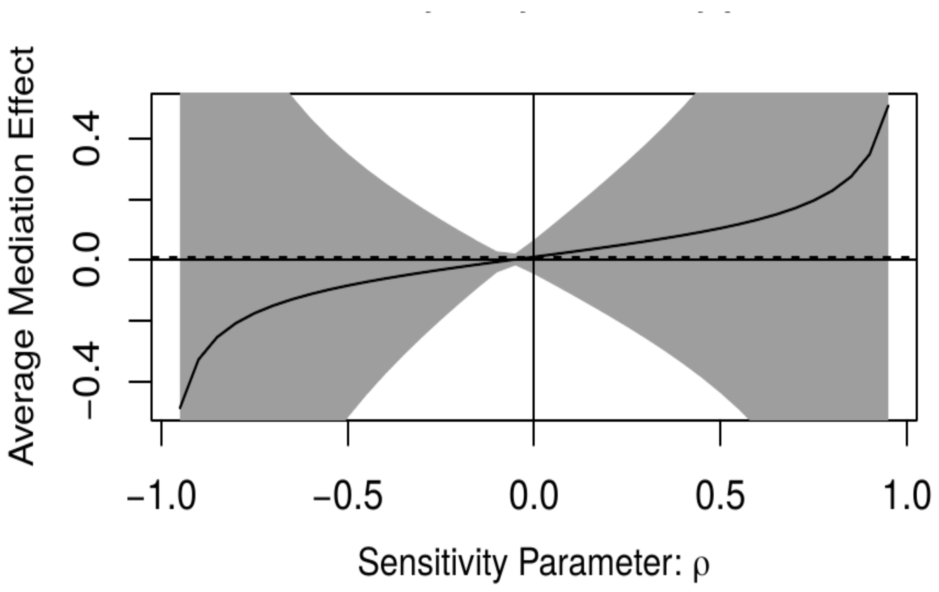

Figure S10 Overall sensitivity of the mediating pathways for the four dietary indices

Note:All four dietary indices (E-DII13, MAR, DBI-LBS, CHEI-9) demonstrated unstable mediating pathways, which corroborates the finding of no significant mediation effects when using only one mediator variable.

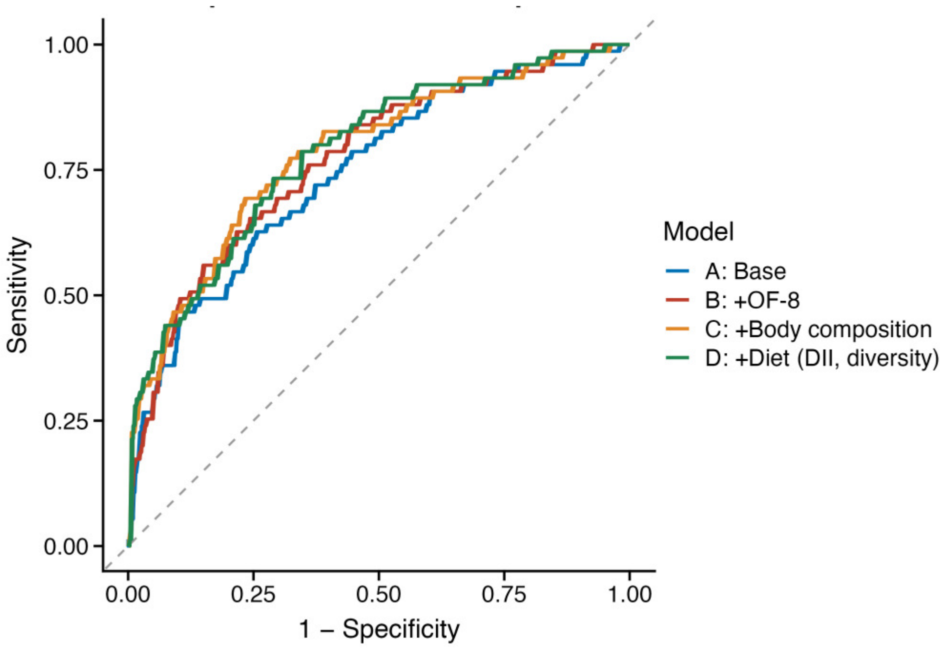

Figure S11 Discriminative performance of nested prediction models for poor health

Note:Receiver operating characteristic curves comparing the discrimination ability of sequentially nested models for predicting poor health, including the baseline demographic model, the OF-8-enhanced model, the body composition model, and the full model incorporating dietary indicators.

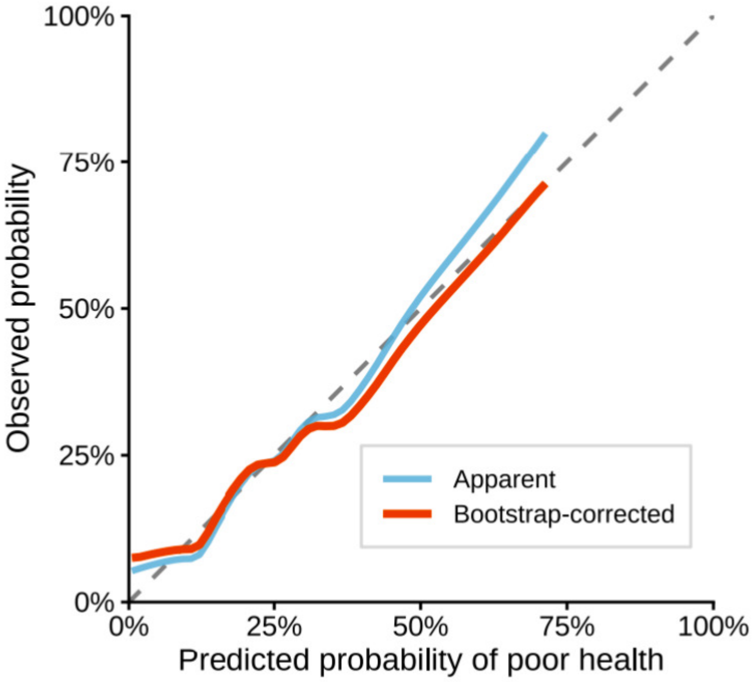

Figure S12 Calibration of the final prediction model for poor health

Note:Calibration plot showing the agreement between predicted and observed probabilities of poor health. Apparent and bootstrap-corrected calibration curves are presented to assess potential optimism in model performance.The calibrated calibration curve after Bootstrap adjustment shows a slope of 0.730 and an intercept of approximately 0, indicating that Model D exhibits somewhat overly optimistic predictions in high-risk segments, but the overall calibration remains acceptable.

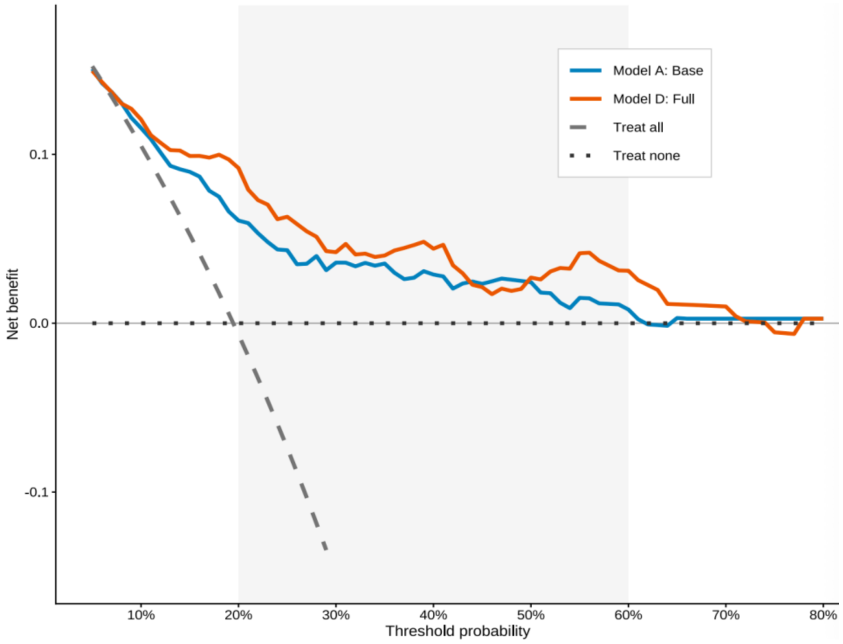

Figure S13 Decision curve analysis of prediction models for poor health

Note:Decision curve analysis comparing the clinical net benefit of the baseline model and the full prediction model across a range of threshold probabilities, with treat-all and treat-none strategies shown as references.

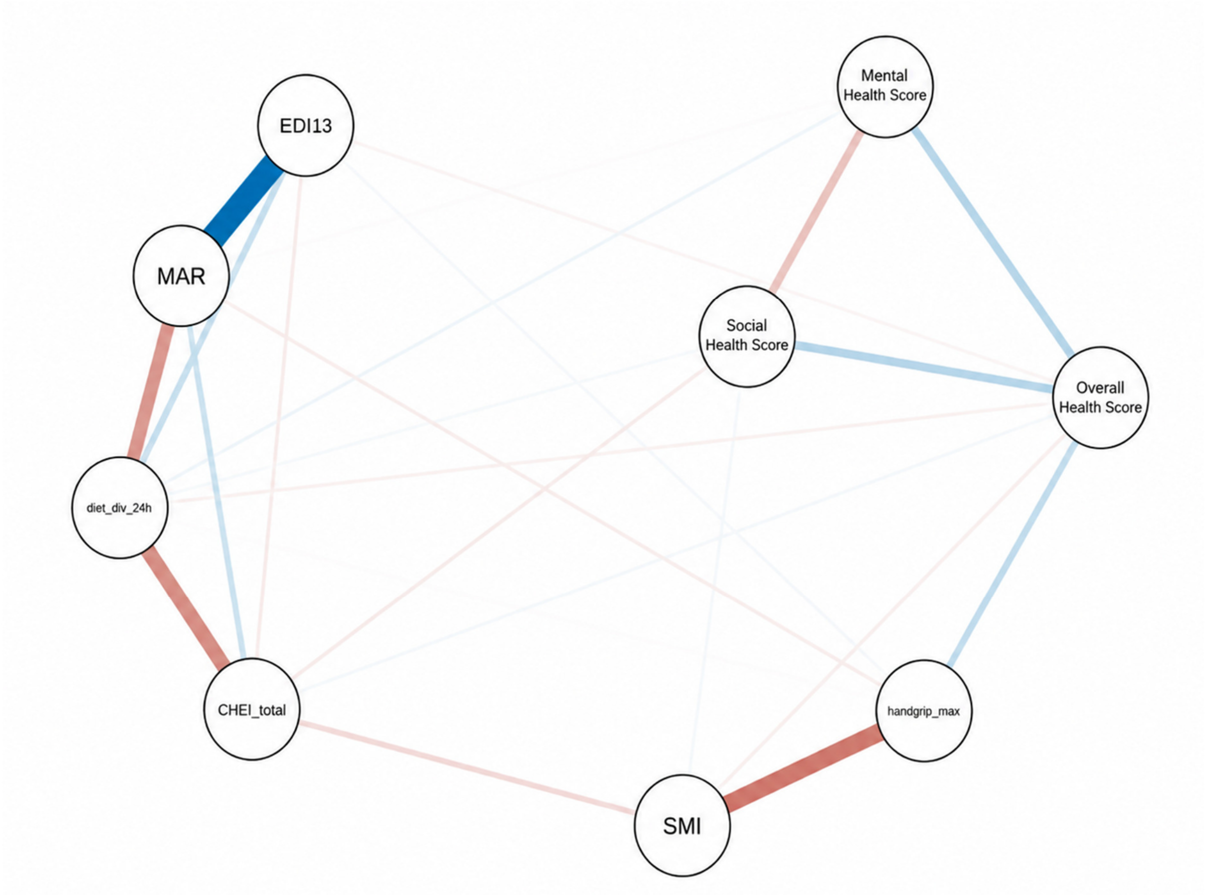

Figure S14 Bayesian network based on score-based search

Note:Dietary diversity, grip strength, and physical health are positioned as core nodes; the visualization of inter-node relationships reveals complex dependency structures that the linear mediation model failed to capture.

S4 STROBE Cross-sectional Study Research Report List

The table below indicates the location of each item from the 22-item STROBE (Strengthening the Reporting of Observational Studies in Epidemiology) checklist within the main text; a new statement "This study adheres to the STROBE guidelines for cross-sectional studies" has been added in the Methods section.

Table STROBE List

Term	Recommendation Entry	Location
1a	The title specifies the study design (cross-sectional).	Title
1b	The abstract provides a balanced and comprehensive summary.	Abstract
2	Introduction – Scientific Background and Basis	Introduction
3	Introduction – Defining Research Objectives and Hypotheses	Introduction

Term	Recommendation Entry	Location
4	Method – Description of Research Design	2.1
5	Research Location (Time, Place)	2.1
6	Participants were excluded based on the specified criteria and methodology.	2.1、 S1.2
7	Variable definitions (exposure, outcome, confounding factors, effect modifiers)	2.2-2.5、 S1.3-S1.7
8	Data Sources and Measurement Methods	2.3-2.5、S1.5-S1.6
9	Bias control (IPTW, negative control, E-value)	2.5、 S1.8
10	Sample Size Calculation	S1.1
11	Continuous variable processing (z-standardization, RCS, etc.)	2.5、 S1.8
12a	Statistical Methods (Main Analysis)	2.5
12b	Subgroup and Interaction Analysis	2.5
12c	Missing Data Processing	2.5、 S1.8
12d	Study Population Description (Sampling Method)	2.1、 S1.1
13	Number of Participants (Flowchart)	Figure1
14	Descriptive Data	3.1、Table 1、S2
15	Event Outcome (Health Grade Distribution)	3.1、S2
16	Main Results (Unadjusted vs. Adjusted Models)	3.2、Table 2、 S2
17	Other analyses (sensitivity, subgroups)	3.3-3.4、 S2
18	Discussion – Key Results Summary	4
19	Discussion – Limitations	4
20	Discussion – Result Interpretation	4
21	Discussion – Extrapolation	4
22	Sources of funds	Funding Statement

S5 Supplementary References

[S1] Tanaka T, Takahashi K, Hirano H, et al. Oral frailty as a risk factor for physical frailty and mortality in community-dwelling elderly. J Gerontol A Biol Sci Med Sci. 2018;73(12):1661-1667.

[S2] Shivappa N, Steck SE, Hurley TG, Hussey JR, Hébert JR. Designing and developing a literature-derived, population-based dietary inflammatory index. Public Health Nutr. 2014;17(8):1689-1696.

[S3] National Health Commission. WS/T 802—2022 Health Assessment for the Elderly [S]. Beijing: China Standards Press, 2022.

[S4] China Nutrition Society. Dietary Guidelines for Chinese Residents (2022) [M]. Beijing: People's Medical Publishing House, 2022.

[S5] Yang Yuexin, Wang Guangya, Pan Xingchang. China Food Composition Table (Standard Edition, 6th

Edition) [M]. Beijing: Peking University Medical Press, 2018.

[S6] VanderWeele TJ, Ding P. Sensitivity analysis in observational research: introducing the E-value. Ann Intern Med. 2017;167(4):268-274.

[S7] Imai K, Keele L, Tingley D. A general approach to causal mediation analysis. Psychol Methods. 2010;15(4):309-334.

[S8] DeLong ER, DeLong DM, Clarke-Pearson DL. Comparing the areas under two or more correlated receiver operating characteristic curves: a nonparametric approach. Biometrics. 1988;44(3):837-845.

[S9] Harrell FE Jr. Regression Modeling Strategies. 2nd ed. New York: Springer; 2015.

[S10] von Elm E, Altman DG, Egger M, et al. The Strengthening the Reporting of Observational Studies in Epidemiology (STROBE) statement: guidelines for reporting observational studies. Lancet. 2007;370(9596):1453-1457.
