# Supplementary material for: Oral Frailty and Multidimensional Health Among Community-Dwelling Older Adults in China: A Cross-Sectional Study
Source: Nutrients. 2026 Jul 9;18(14):2250. doi: 10.3390/nu18142250 (PMC13414565; doi:10.3390/nu18142250)
Supplement: Supplementary file 1 [file nutrients-18-02250-s001.zip › TableS1_fulltable_baseline_OF8.pdf]

Table S1 Baseline Characteristics According to OF-8-Defined Oral Frailty Status

Variable	Level	Overall	No oral frailty	Oral frailty	*P*	SMD
n		454	186	268		
Age_body (mean (SD))		70.82 (7.48)	69.19 (7.08)	71.95 (7.55)	<0.001	0.376
Age_group (%)	60-69	178 (40.9)	82 (46.9)	96 (36.9)	0.032	0.262
	70-79	195 (44.8)	76 (43.4)	119 (45.8)		
	≥80	62 (14.3)	17 (9.7)	45 (17.3)		
Sex_body (%)	Female	310 (68.3)	128 (68.8)	182 (67.9)	0.919	0.019
	Male	144 (31.7)	58 (31.2)	86 (32.1)		
Education (%)	High school or vocational secondary school	263 (58.1)	102 (54.8)	161 (60.3)	0.440	0.156
	Primary school	104 (23.0)	47 (25.3)	57 (21.3)		
	Below primary school	59 (13.0)	23 (12.4)	36 (13.5)		
	Undergraduate degree or higher	27 (6.0)	14 (7.5)	13 (4.9)		
Family income (%)	＜30000¥	223 (51.9)	94 (53.7)	129 (50.6)	0.776	0.128
	＞90000¥	3 (0.7)	2 (1.1)	1 (0.4)		
	30000-50000¥	162 (37.7)	63 (36.0)	99 (38.8)		
	50000-70000¥	36 (8.4)	13 (7.4)	23 (9.0)		
	70000-90000¥	6 (1.4)	3 (1.7)	3 (1.2)		
BMI (mean (SD))		22.82 (3.42)	23.35 (3.41)	22.44 (3.39)	0.006	0.266
Body_fat_pct (mean (SD))		25.40 (8.74)	26.15 (8.78)	24.87 (8.69)	0.128	0.147
SMI (mean (SD))		15.88 (1.74)	16.08 (1.80)	15.73 (1.69)	0.034	0.204
Handgrip_max (mean (SD))		22.29 (11.80)	23.72 (12.71)	21.32 (11.06)	0.045	0.201
SBP (mean (SD))		132.90 (19.47)	133.29 (20.16)	132.64 (19.03)	0.742	0.033
DBP (mean (SD))		79.15 (10.67)	81.16 (11.25)	77.81 (10.06)	0.002	0.314
HR (mean (SD))		79.99 (11.64)	80.52 (11.07)	79.64 (12.02)	0.457	0.076
Ill_hypertension (%)	No	274 (60.4)	117 (62.9)	157 (58.6)	0.408	0.089
	Yes	180 (39.6)	69 (37.1)	111 (41.4)		
Ill_diabetes (%)	No	386 (85.0)	167 (89.8)	219 (81.7)	0.025	0.232
	Yes	68 (15.0)	19 (10.2)	49 (18.3)		
Ill_hyperlipemia (%)	No	326 (71.8)	145 (78.0)	181 (67.5)	0.020	0.236
	Yes	128 (28.2)	41 (22.0)	87 (32.5)		

Variable	Level	Overall	No oral frailty	Oral frailty	*P*	SMD
Ill_heart (%)	No	365 (80.4)	161 (86.6)	204 (76.1)	0.008	0.270
	Yes	89 (19.6)	25 (13.4)	64 (23.9)		
Chronic disease_count (mean (SD))		1.02 (1.05)	0.83 (0.97)	1.16 (1.07)	0.001	0.325
Smoke (%)	Never	373 (82.2)	154 (82.8)	219 (81.7)	0.421	0.162
	In the past 30 days has	13 (2.9)	7 (3.8)	6 (2.2)		
	Smoke-Free	31 (6.8)	9 (4.8)	22 (8.2)		
	Frequently	37 (8.1)	16 (8.6)	21 (7.8)		
Alcohol (%)	Never	317 (69.8)	135 (72.6)	182 (67.9)	0.659	0.122
	In the past 30 days has	53 (11.7)	18 (9.7)	35 (13.1)		
	Alcohol-Free	50 (11.0)	19 (10.2)	31 (11.6)		
	Frequently	34 (7.5)	14 (7.5)	20 (7.5)		
Areca(%)	Never	442 (97.4)	182 (97.8)	260 (97.0)	0.344	0.141
	In the past 30 days has	3 (0.7)	2 (1.1)	1 (0.4)		
	Areca-Free	9 (2.0)	2 (1.1)	7 (2.6)		
Weekly_exercise (%)	never	142 (31.3)	56 (30.1)	86 (32.1)	0.455	0.183
	1-2days/week	36 (7.9)	11 (5.9)	25 (9.3)		
	3-4days/week	25 (5.5)	10 (5.4)	15 (5.6)		
	5-6days/week	24 (5.3)	13 (7.0)	11 (4.1)		
	everyday	227 (50.0)	96 (51.6)	131 (48.9)		
Vegetable (%)	＜1/day	8 (1.8)	4 (2.2)	4 (1.5)	0.092	0.253
	≥3/day	164 (36.5)	73 (39.7)	91 (34.3)		
	1/day	33 (7.3)	7 (3.8)	26 (9.8)		
	2/day	244 (54.3)	100 (54.3)	144 (54.3)		
Fruit (%)	1-2/week	45 (10.0)	23 (12.5)	22 (8.3)	0.001	0.451
	3-5/week	62 (13.8)	22 (12.0)	40 (15.1)		
	everyday	276 (61.5)	127 (69.0)	149 (56.2)		
	≤1/month	29 (6.5)	5 (2.7)	24 (9.1)		
	2-3/month	37 (8.2)	7 (3.8)	30 (11.3)		
Milk (%)	1-2/week	39 (8.7)	16 (8.7)	23 (8.7)	0.834	0.116
	3-5/week	39 (8.7)	18 (9.8)	21 (8.0)		
	everyday	167 (37.4)	66 (35.9)	101 (38.4)		
	≤1/month	152 (34.0)	66 (35.9)	86 (32.7)		
	2-3/month	50 (11.2)	18 (9.8)	32 (12.2)		
Sea food (%)	1-2/week	79 (17.6)	26 (14.2)	53 (20.0)	0.103	0.263

Variable	Level	Overall	No oral frailty	Oral frailty	*P*	SMD
	3-5/week	19 (4.2)	12 (6.6)	7 (2.6)		
	everyday	8 (1.8)	5 (2.7)	3 (1.1)		
	≤1/month	200 (44.6)	80 (43.7)	120 (45.3)		
	2-3/month	142 (31.7)	60 (32.8)	82 (30.9)		
Ecal (mean (SD))		1303.90 (1828.46)	1257.18 (727.30)	1336.42 (2304.23)	0.655	0.046
Prot (mean (SD))		38.59 (26.14)	39.63 (26.10)	37.87 (26.20)	0.488	0.067
Fiber (mean (SD))		5.55 (5.51)	5.99 (6.06)	5.24 (5.09)	0.159	0.135
EDII13 (mean (SD))		1.75 (0.64)	1.73 (0.66)	1.76 (0.62)	0.722	0.034
MAR (mean (SD))		44.74 (20.05)	45.45 (20.90)	44.24 (19.45)	0.532	0.060
Prot_density (mean (SD))		31.85 (11.87)	31.61 (11.34)	32.01 (12.24)	0.723	0.035
Energy_adequacy (mean (SD))		84.51 (101.17)	80.12 (48.38)	87.62 (125.82)	0.449	0.079
Diet_div_ffq (mean (SD))		13.41 (2.68)	13.71 (2.66)	13.21 (2.68)	0.054	0.188
Diet_div_24h (mean (SD))		5.67 (1.51)	5.76 (1.58)	5.60 (1.46)	0.296	0.103
CHEI_total (mean (SD))		42.72 (11.06)	42.05 (11.59)	43.19 (10.67)	0.294	0.102
NRF_simple (mean (SD))		131.75 (101.38)	122.09 (98.40)	138.55 (103.08)	0.099	0.163
NaK_ratio (mean (SD))		0.44 (0.54)	0.40 (0.40)	0.46 (0.62)	0.212	0.125
AOXI (mean (SD))		0.00 (3.61)	0.03 (3.54)	-0.02 (3.67)	0.879	0.015
DBI_HBS (mean (SD))		13.73 (6.58)	13.80 (6.52)	13.69 (6.63)	0.868	0.016
DBI_LBS (mean (SD))		32.07 (7.74)	31.73 (8.10)	32.30 (7.49)	0.447	0.073
DBI_DQD (mean (SD))		45.80 (8.79)	45.52 (9.55)	45.99 (8.24)	0.586	0.052
Vegetable_g (mean (SD))		209.97 (226.50)	223.01 (253.28)	200.93 (205.93)	0.314	0.096
Fruit_g (mean (SD))		198.49 (531.44)	199.01 (242.91)	198.13 (661.96)	0.986	0.002
Dairy_g (mean (SD))		52.77 (116.66)	51.45 (118.74)	53.68 (115.41)	0.843	0.019
Soybean_g (mean (SD))		8.71 (41.22)	7.85 (41.27)	9.32 (41.26)	0.712	0.036
Red_meat_g (mean (SD))		50.12 (77.41)	55.71 (89.23)	46.24 (67.93)	0.206	0.120
Fish_g (mean (SD))		16.73 (53.76)	15.81 (52.01)	17.37 (55.03)	0.765	0.029
Egg_g (mean (SD))		33.97 (43.45)	32.42 (43.69)	35.05 (43.33)	0.532	0.060
Cooking_oil_g (mean (SD))		26.58 (21.48)	29.30 (26.44)	24.70 (17.03)	0.027	0.207
Tooth_number (mean (SD))		22.09 (8.80)	23.83 (7.87)	20.94 (9.21)	0.001	0.337
OHCI (mean (SD))		2.47 (1.36)	2.38 (1.40)	2.54 (1.33)	0.204	0.121
Probable_sarc (%)	FALSE	206 (51.6)	95 (58.6)	111 (46.8)	0.027	0.238
	TRUE	193 (48.4)	67 (41.4)	126 (53.2)		
Overall health score (mean (SD))		85.66 (9.74)	87.41 (8.89)	84.44 (10.13)	0.001	0.312
Body health score (mean (SD))		44.26 (5.37)	45.48 (4.55)	43.40 (5.73)	<0.001	0.402
Mental health score (mean (SD))		27.98 (2.93)	28.24 (2.30)	27.80 (3.29)	0.118	0.154
Social health score (mean (SD))		13.43 (4.54)	13.69 (4.86)	13.24 (4.30)	0.294	0.099
Overall health (%)	unhealthy	90 (19.8)	20 (10.8)	70 (26.1)	<0.001	0.423
	basic healthy	217 (47.8)	93 (50.0)	124 (46.3)		

Variable	Level	Overall	No oral frailty	Oral frailty	*P*	SMD
	healthy	147 (32.4)	73 (39.2)	74 (27.6)		
Physical health (%)	unhealthy	83 (18.3)	17 (9.1)	66 (24.6)	<0.001	0.427
	basic healthy	15 (3.3)	8 (4.3)	7 (2.6)		
	healthy	356 (78.4)	161 (86.6)	195 (72.8)		
Mental health (%)	unhealthy	17 (3.7)	3 (1.6)	14 (5.2)	0.094	0.216
	basic healthy	11 (2.4)	6 (3.2)	5 (1.9)		
	healthy	426 (93.8)	177 (95.2)	249 (92.9)		
Social health (%)	unhealthy	119 (26.2)	44 (23.7)	75 (28.0)	0.098	0.206
	basic healthy	164 (36.1)	61 (32.8)	103 (38.4)		
	healthy	171 (37.7)	81 (43.5)	90 (33.6)		
