# Supplementary material for: Oral Frailty and Multidimensional Health Among Community-Dwelling Older Adults in China: A Cross-Sectional Study
Source: Nutrients. 2026 Jul 9;18(14):2250. doi: 10.3390/nu18142250 (PMC13414565; doi:10.3390/nu18142250)
Supplement: Supplementary file 1 [file nutrients-18-02250-s001.zip › TableS2_fulltable_baseline_SOFT6.pdf]

Table S2 Baseline Characteristics According to SOFT-6-Defined Oral Frailty Status

Variable	Level	Overall	No oral frailty	Oral frailty	*P*	SMD
n		454	216	238		
Age_body (mean (SD))		70.82 (7.48)	69.73 (6.97)	71.81 (7.79)	0.003	0.281
Age_group (%)	60-69	178 (40.9)	94 (45.6)	84 (36.7)	0.035	0.251
	70-79	195 (44.8)	91 (44.2)	104 (45.4)		
	≥80	62 (14.3)	21 (10.2)	41 (17.9)		
Sex_body (%)	Female	310 (68.3)	150 (69.4)	160 (67.2)	0.685	0.048
	Male	144 (31.7)	66 (30.6)	78 (32.8)		
Education (%)	High school or vocational secondary school	263 (58.1)	137 (63.4)	126 (53.2)	0.052	0.264
	Primary school	104 (23.0)	39 (18.1)	65 (27.4)		
	Below primary school	59 (13.0)	25 (11.6)	34 (14.3)		
	Undergraduate degree or higher	27 (6.0)	15 (6.9)	12 (5.1)		
Family income (%)	＜30000¥	223 (51.9)	100 (49.3)	123 (54.2)	0.729	0.138
	＞90000¥	3 (0.7)	2 (1.0)	1 (0.4)		
	30000-50000¥	162 (37.7)	78 (38.4)	84 (37.0)		
	50000-70000¥	36 (8.4)	20 (9.9)	16 (7.0)		
	70000-90000¥	6 (1.4)	3 (1.5)	3 (1.3)		
BMI (mean (SD))		22.82 (3.42)	23.17 (3.25)	22.49 (3.55)	0.037	0.199
Body_fat_pct (mean (SD))		25.40 (8.74)	26.12 (8.41)	24.73 (9.00)	0.094	0.159
SMI (mean (SD))		15.88 (1.74)	15.98 (1.70)	15.78 (1.78)	0.222	0.116
Handgrip_max (mean (SD))		22.29 (11.80)	23.40 (12.42)	21.25 (11.11)	0.067	0.182
SBP (mean (SD))		132.90 (19.47)	131.47 (19.17)	134.22 (19.70)	0.156	0.141
DBP (mean (SD))		79.15 (10.67)	79.14 (10.71)	79.16 (10.66)	0.987	0.002
HR (mean (SD))		79.99 (11.64)	79.68 (11.68)	80.29 (11.62)	0.604	0.052
Ill_hypertension (%)	No	274 (60.4)	138 (63.9)	136 (57.1)	0.170	0.138
	Yes	180 (39.6)	78 (36.1)	102 (42.9)		
Ill_diabetes (%)	No	386 (85.0)	196 (90.7)	190 (79.8)	0.002	0.312
	Yes	68 (15.0)	20 (9.3)	48 (20.2)		
Ill_hyperlipemia (%)	No	326 (71.8)	156 (72.2)	170 (71.4)	0.934	0.018
	Yes	128 (28.2)	60 (27.8)	68 (28.6)		
Ill_heart (%)	No	365 (80.4)	181 (83.8)	184 (77.3)	0.105	0.164
	Yes	89 (19.6)	35 (16.2)	54 (22.7)		
Chronic disease_count (mean (SD))		1.02 (1.05)	0.89 (0.97)	1.14 (1.10)	0.011	0.241

Variable	Level	Overall	No oral frailty	Oral frailty	*P*	SMD
Smoke (%)	Never	373 (82.2)	182 (84.3)	191 (80.3)	0.544	0.138
	In the past 30 days has	13 (2.9)	7 (3.2)	6 (2.5)		
	Smoke-Free	31 (6.8)	12 (5.6)	19 (8.0)		
	Frequently	37 (8.1)	15 (6.9)	22 (9.2)		
Alcohol (%)	Never	317 (69.8)	158 (73.1)	159 (66.8)	0.048	0.268
	In the past 30 days has	53 (11.7)	24 (11.1)	29 (12.2)		
	Alcohol-Free	50 (11.0)	15 (6.9)	35 (14.7)		
	Frequently	34 (7.5)	19 (8.8)	15 (6.3)		
Areca(%)	Never	442 (97.4)	210 (97.2)	232 (97.5)	0.789	0.064
	In the past 30 days has	3 (0.7)	2 (0.9)	1 (0.4)		
	Areca-Free	9 (2.0)	4 (1.9)	5 (2.1)		
Weekly_exercise (%)	never	142 (31.3)	65 (30.1)	77 (32.4)	0.147	0.248
	1-2days/week	36 (7.9)	11 (5.1)	25 (10.5)		
	3-4days/week	25 (5.5)	10 (4.6)	15 (6.3)		
	5-6days/week	24 (5.3)	13 (6.0)	11 (4.6)		
	everyday	227 (50.0)	117 (54.2)	110 (46.2)		
Vegetable (%)	＜1/day	8 (1.8)	3 (1.4)	5 (2.1)	0.306	0.181
	≥3/day	164 (36.5)	83 (39.2)	81 (34.2)		
	1/day	33 (7.3)	11 (5.2)	22 (9.3)		
	2/day	244 (54.3)	115 (54.2)	129 (54.4)		
Fruit (%)	1-2/week	45 (10.0)	21 (9.9)	24 (10.1)	0.008	0.359
	3-5/week	62 (13.8)	28 (13.2)	34 (14.3)		
	everyday	276 (61.5)	145 (68.4)	131 (55.3)		
	≤1/month	29 (6.5)	8 (3.8)	21 (8.9)		
	2-3/month	37 (8.2)	10 (4.7)	27 (11.4)		
Milk (%)	1-2/week	39 (8.7)	21 (9.9)	18 (7.7)	0.791	0.124
	3-5/week	39 (8.7)	19 (9.0)	20 (8.5)		
	everyday	167 (37.4)	82 (38.7)	85 (36.2)		
	≤1/month	152 (34.0)	69 (32.5)	83 (35.3)		
	2-3/month	50 (11.2)	21 (9.9)	29 (12.3)		
Sea food (%)	1-2/week	79 (17.6)	38 (18.0)	41 (17.3)	0.722	0.137
	3-5/week	19 (4.2)	10 (4.7)	9 (3.8)		
	everyday	8 (1.8)	4 (1.9)	4 (1.7)		
	≤1/month	200 (44.6)	87 (41.2)	113 (47.7)		
	2-3/month	142 (31.7)	72 (34.1)	70 (29.5)		
Ecal (mean (SD))		1303.90 (1828.46)	1401.09 (2528.28)	1215.54 (755.51)	0.288	0.099
Prot (mean (SD))		38.59 (26.14)	40.18 (27.13)	37.14 (25.19)	0.222	0.116

Variable	Level	Overall	No oral frailty	Oral frailty	*P*	SMD
Fiber (mean (SD))		5.55 (5.51)	5.72 (5.66)	5.39 (5.38)	0.536	0.059
EDII13 (mean (SD))		1.75 (0.64)	1.74 (0.63)	1.75 (0.64)	0.915	0.010
MAR (mean (SD))		44.74 (20.05)	45.92 (20.61)	43.67 (19.50)	0.239	0.112
Prot_density (mean (SD))		31.85 (11.87)	32.17 (11.46)	31.55 (12.25)	0.584	0.052
Energy_adequacy (mean (SD))		84.51 (101.17)	88.27 (136.85)	80.99 (47.95)	0.455	0.071
Diet_div_ffq (mean (SD))		13.41 (2.68)	13.79 (2.60)	13.07 (2.71)	0.005	0.269
Diet_div_24h (mean (SD))		5.67 (1.51)	5.84 (1.54)	5.50 (1.46)	0.019	0.230
CHEI_total (mean (SD))		42.72 (11.06)	42.82 (11.30)	42.62 (10.86)	0.848	0.019
NRF_simple (mean (SD))		131.75 (101.38)	129.13 (97.10)	134.18 (105.36)	0.608	0.050
NaK_ratio (mean (SD))		0.44 (0.54)	0.46 (0.53)	0.42 (0.55)	0.407	0.079
AOXI (mean (SD))		0.00 (3.61)	0.25 (3.91)	-0.23 (3.31)	0.163	0.133
DBI_HBS (mean (SD))		13.73 (6.58)	14.20 (6.51)	13.31 (6.62)	0.160	0.134
DBI_LBS (mean (SD))		32.07 (7.74)	31.64 (7.83)	32.45 (7.65)	0.270	0.105
DBI_DQD (mean (SD))		45.80 (8.79)	45.83 (9.18)	45.77 (8.44)	0.937	0.007
Vegetable_g (mean (SD))		209.97 (226.50)	203.01 (238.53)	216.27 (215.36)	0.540	0.058
Fruit_g (mean (SD))		198.49 (531.44)	241.39 (730.68)	159.66 (230.09)	0.106	0.151
Dairy_g (mean (SD))		52.77 (116.66)	53.20 (114.53)	52.38 (118.80)	0.941	0.007
Soybean_g (mean (SD))		8.71 (41.22)	6.86 (38.40)	10.40 (43.64)	0.368	0.086
Red_meat_g (mean (SD))		50.12 (77.41)	52.96 (83.93)	47.55 (71.08)	0.463	0.070
Fish_g (mean (SD))		16.73 (53.76)	17.05 (49.62)	16.45 (57.36)	0.907	0.011
Egg_g (mean (SD))		33.97 (43.45)	38.52 (46.20)	29.85 (40.46)	0.036	0.200
Cooking_oil_g (mean (SD))		26.58 (21.48)	27.52 (21.66)	25.73 (21.33)	0.383	0.083
Tooth_number (mean (SD))		22.09 (8.80)	24.27 (7.47)	20.07 (9.46)	<0.001	0.493
OHCI (mean (SD))		2.47 (1.36)	2.44 (1.35)	2.51 (1.37)	0.567	0.054
Probable_sarc (%)	FALSE	206 (51.6)	114 (58.5)	92 (45.1)	0.010	0.270
	TRUE	193 (48.4)	81 (41.5)	112 (54.9)		
Overall health score (mean (SD))		85.66 (9.74)	88.08 (7.29)	83.46 (11.09)	<0.001	0.492
Body health score (mean (SD))		44.26 (5.37)	45.69 (3.88)	42.95 (6.16)	<0.001	0.533
Mental health score (mean (SD))		27.98 (2.93)	28.56 (1.87)	27.45 (3.56)	<0.001	0.394
Social health score (mean (SD))		13.43 (4.54)	13.82 (4.37)	13.07 (4.66)	0.078	0.166
Overall health (%)	unhealthy	90 (19.8)	24 (11.1)	66 (27.7)	<0.001	0.445
	basic healthy	217 (47.8)	109 (50.5)	108 (45.4)		
	healthy	147 (32.4)	83 (38.4)	64 (26.9)		
Physical health (%)	unhealthy	83 (18.3)	23 (10.6)	60 (25.2)	<0.001	0.434
	basic healthy	15 (3.3)	4 (1.9)	11 (4.6)		
	healthy	356 (78.4)	189 (87.5)	167 (70.2)		
Mental health (%)	unhealthy	17 (3.7)	2 (0.9)	15 (6.3)	0.001	0.354
	basic healthy	11 (2.4)	2 (0.9)	9 (3.8)		

**Variable**	**Level**	**Overall**	**No oral frailty**	**Oral frailty**	***P***	**SMD**
	healthy	426 (93.8)	212 (98.1)	214 (89.9)		
Social health (%)	unhealthy	119 (26.2)	53 (24.5)	66 (27.7)	0.173	0.177
	basic healthy	164 (36.1)	72 (33.3)	92 (38.7)		
	healthy	171 (37.7)	91 (42.1)	80 (33.6)		
